# Soaking suggests “alternative facts”: Only co-crystallization discloses major ligand-induced interface rearrangements of a homodimeric tRNA-binding protein indicating a novel mode-of-inhibition

**DOI:** 10.1371/journal.pone.0175723

**Published:** 2017-04-18

**Authors:** Frederik Rainer Ehrmann, Johann Stojko, Alexander Metz, François Debaene, Luzi Jakob Barandun, Andreas Heine, François Diederich, Sarah Cianférani, Klaus Reuter, Gerhard Klebe

**Affiliations:** 1 Institut für Pharmazeutische Chemie, Philipps-Universität Marburg, Marburg, Germany; 2 Laboratoire de Spectrométrie de Masse Bio-Organique, Université de Strasbourg, CNRS, IPHC UMR 7178, Strasbourg, France; 3 Laboratorium für Organische Chemie, ETH Zurich, Zurich, Switzerland; Griffith University, AUSTRALIA

## Abstract

For the efficient pathogenesis of *Shigella*, the causative agent of bacillary dysentery, full functionality of tRNA-guanine transglycosylase (TGT) is mandatory. TGT performs post-transcriptional modifications of tRNAs in the anticodon loop taking impact on virulence development. This suggests TGT as a putative target for selective anti-shigellosis drug therapy. Since bacterial TGT is only functional as homodimer, its activity can be inhibited either by blocking its active site or by preventing dimerization. Recently, we discovered that in some crystal structures obtained by soaking the full conformational adaptation most likely induced in solution upon ligand binding is not displayed. Thus, soaked structures may be misleading and suggest irrelevant binding modes. Accordingly, we re-investigated these complexes by co-crystallization. The obtained structures revealed large conformational rearrangements not visible in the soaked complexes. They result from spatial perturbations in the ribose-34/phosphate-35 recognition pocket and, consequently, an extended *loop-helix motif* required to prevent access of water molecules into the dimer interface loses its geometric integrity. Thermodynamic profiles of ligand binding in solution indicate favorable entropic contributions to complex formation when large conformational adaptations in the dimer interface are involved. Native MS titration experiments reveal the extent to which the homodimer is destabilized in the presence of each inhibitor. Unexpectedly, one ligand causes a complete rearrangement of subunit packing within the homodimer, never observed in any other TGT crystal structure before. Likely, this novel *twisted dimer* is catalytically inactive and, therefore, suggests that stabilizing this non-productive subunit arrangement may be used as a further strategy for TGT inhibition.

## Introduction

Bacterial tRNA-guanine transglycosylase (TGT; EC 2.4.2.29) catalyzes the exchange of the genetically encoded guanine-34 in the wobble position of tRNAs^Asp,Asn,His,Tyr^ by the pre-modified base preQ_1_ (7-aminomethyl-7-deazaguanine) [1]. At the level of tRNA, this base is then further modified to queuine [2, 3]. In *Shigella* spp., the causative agents of bacillary dysentery, TGT function is essential for the efficient translation of *virF* mRNA encoding a transcriptional activator, which in turn is required for the expression of a large number of pathogenicity genes [4]. Inactivation of the *tgt* gene results in a significantly weakened virulence phenotype suggesting TGT as a putative target for the rational design of anti-shigellosis compounds [5, 6]. Bacillary dysentery or shigellosis is a severe diarrheal disease [7] with approximately 150 million cases causing more than 70.000 fatalities per year [8, 9]. It mainly occurs in developing countries with substandard hygiene and water supplies [10], but recent reports have also shown increasing incidence in the USA [11]. The emergence of multi-drug resistant *Shigella* strains makes the development of new selective anti-shigellosis compounds a serious need [11–13].

**Fig 1:**
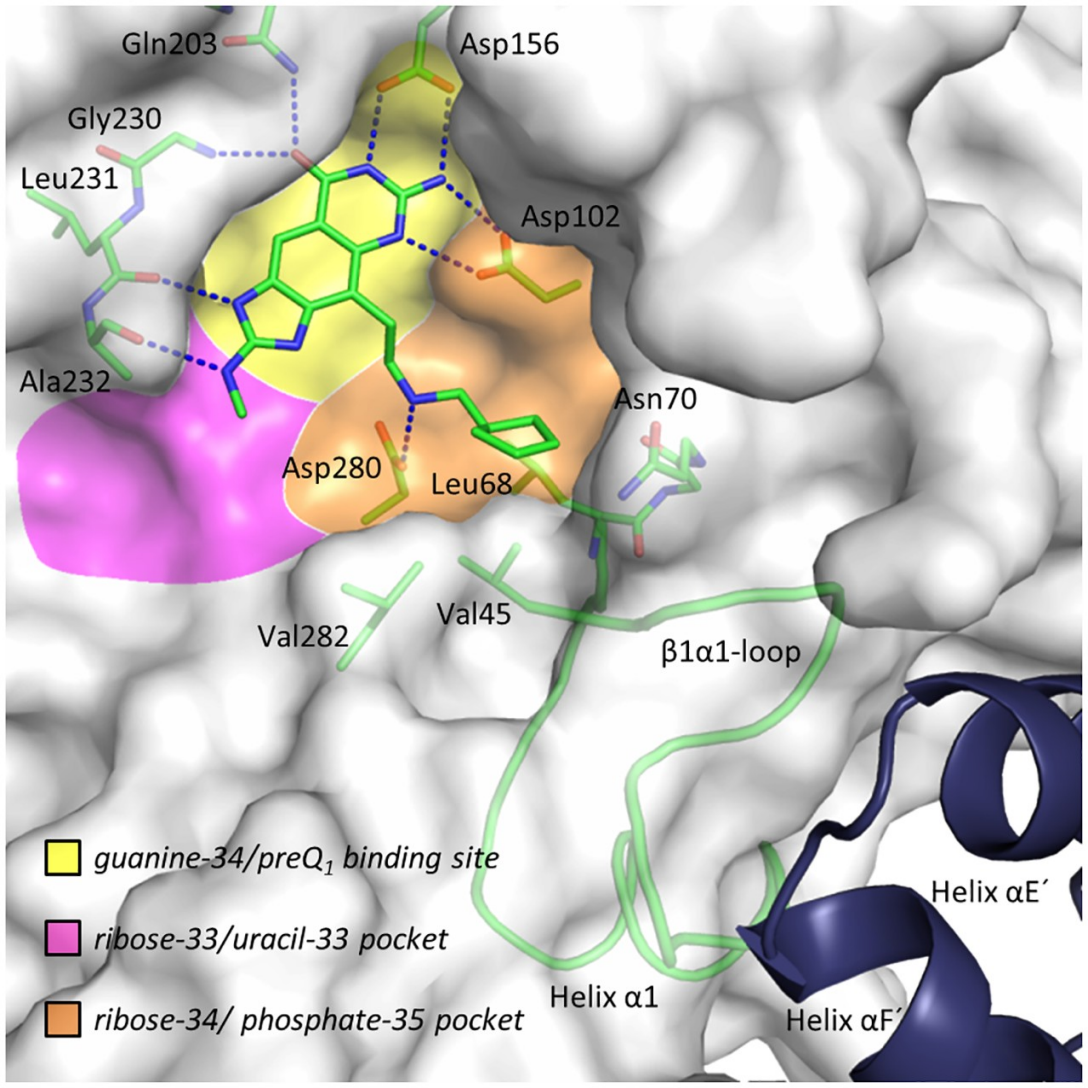
Overview of the active site, its different sub-pockets (yellow, orange, magenta), and part of the second monomer. Co-crystal structure of TGT with inhibitor **5** (TGT∙**5**_co_). The protein is shown as transparent solvent accessible surface and the *loop-helix motif* as cartoon. Color code: C green, O red, N blue. **5** and selected residues are displayed as sticks. For the sake of clarity, residues Tyr106 to Leu111 are not shown. Val45, Leu68 and Val282 form the *hydrophobic floor* of the ribose-34/phosphate-35 pocket (orange). Selected portions of the second monomer of the homodimer are shown as blue cartoon. Blue dashed lines indicate H-bonds from **5** with the protein (2.7–2.9 Å). These characteristics apply to all following figures unless otherwise stated. *N*5 of the *lin*-benzoguanine scaffold was shown to be protonated upon binding to TGT allowing Asp102 to form two H-bonds to the ligand [29].

Since the functional unit of bacterial TGT is a homodimer [14–16], inhibition may not only be achieved *via* compounds blocking its active site but also *via* ligands interfering with dimer formation. In our studies, we use the well crystallizable TGT from *Zymomonas mobilis* as a substitute for TGT from *Shigella spp*. Both enzymes are highly similar and their active sites solely differ by a conservative Tyr106Phe replacement (*Z*. *mobilis* TGT numbering) [17], which was shown to have no significant influence on ligand binding and catalysis [18]. In addition, the key residues involved in interface formation are identical in both species [15,19]. The active site of bacterial TGT comprises three sub-pockets, namely the guanine-34/preQ_1_ binding site, where base exchange occurs, complemented by the adjacent ribose-34/phosphate-35 and ribose-33/uracil-33 recognition sites (Fig 1).

**Fig 2:**
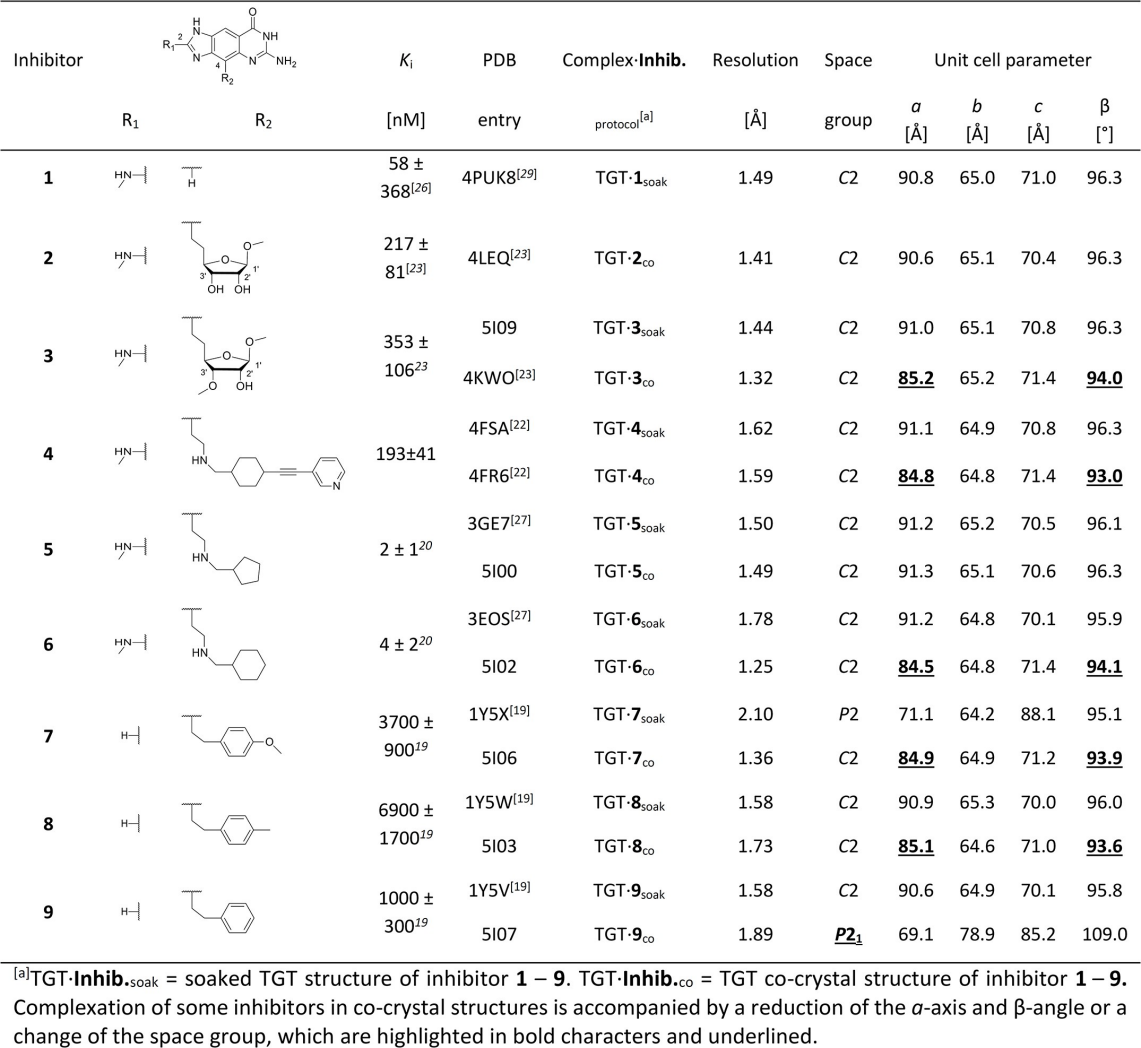
Overview of studied TGT inhibitors, inhibition constants (K_i_), and crystal structures.

**Fig 3:**
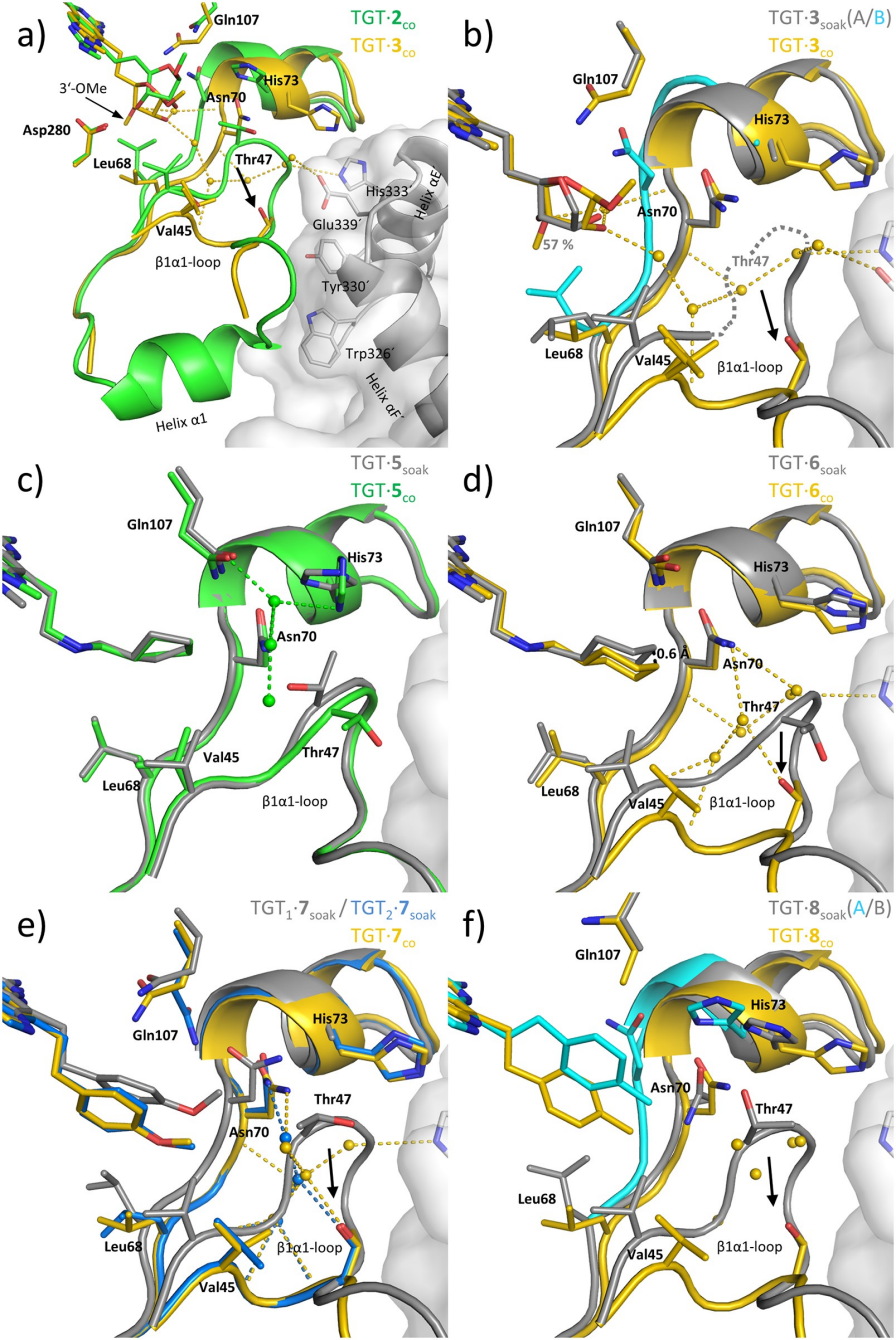
Binding modes and structural arrangements of 2, 3 and 5–8. Color coding for carbons in parenthesis. Comparison of **a)** TGT∙**2**_co_ (green) and TGT∙**3**_co_ (yellow), **b)** TGT∙**3**_soak_ (conformation B cyan and conformation A gray) and TGT∙**3**_co_ (yellow), **c)** TGT∙**5**_soak_ (gray) and TGT∙**5**_co_ (green), **d)** TGT∙**6**_soak_ (gray) and TGT∙**6**_co_ (yellow), **e)** TGT∙**7**_soak_ (TGT_1_ gray; TGT_2_ blue) and TGT∙**7**_co_ (yellow), **f)** TGT∙**8**_soak_ (conformation A: cyan and conformation B gray) and TGT∙**8**_co_ (yellow). Selected water molecules are shown as spheres and colored as the corresponding complex. For the sake of clarity, residues of the guanine-34/preQ_1_ binding site that interact with the *lin*-benzoguanine scaffold are not shown. Solvent accessible surface and selected residues as sticks of the second monomer of the homodimer are colored gray. Position of missing Thr47 in TGT∙**3**_soak_ is indicated as gray dashed lines. Black arrows indicate positional differences between the crystal structures of different crystallization protocols. Occupancies for the different side chains are displayed using the corresponding color of the ligand. Inhibitors **3**, **6**, **7** and **8** displace the *βα*1-loop in the co-crystal structures. Colored dashed lines indicate H-bonds from selected water molecules (2.4–3.5 Å).

In previous studies, we had characterized a number of TGT inhibitors, such as **1**–**9** in Fig 2, which were all based on a *lin*-benzoguanine scaffold endowed with various substituents addressing the adjacent recognition pockets [15, 19–22]. While the *lin*-benzoguanine scaffold invariably occupies the guanine-34/preQ_1_ binding site, its substituents reach into adjacent sub-pockets thereby leading to a broad range of TGT affinities. Recently, we embarked on *lin*-benzoguanines substituted with different furanosides at position 4 [23]. These furanosides were aimed at displacing a conserved water cluster, which, in the presence of the unsubstituted parent *lin*-benzoguanine scaffold, solvates the ribose-34/phosphate-35 pocket (R34/P35-pocket in the following). Among these inhibitors are ligands **2** and **3** (Fig 2), which solely differ by a methyl group attached to the 3´-OH function of the furanoside in **3**. Fitting well into its active site, both ligands inhibit TGT at three-digit nanomolar inhibition constants *K*_i_ with the furanosides mimicking phosphate-35 of a bound tRNA substrate. Surprisingly, co-crystallization of TGT with **3** (but not with **2**) led to crystals with the unit cell *a*-axis reduced by more than 5 Å and the β-angle reduced by about 2° compared to what is normally observed in *Z*. *mobilis* TGT crystals. The resulting crystal structure reveals that the changes of unit cell parameters are accompanied by an altered course of loop *β*1*α*1 in TGT (Fig 3a). In addition, helix *α*1 is no longer crystallographically resolved. These structural elements, hereinafter referred to as *loop-helix motif*, usually shield a cluster of aromatic residues within the TGT homodimer interface from water access [22, 24, 25]. While in our previous work we focused on the interactions of ligands **2** and **3** with active-site residues of bacterial TGT [23], here we investigate their distinct effects on dimer interface architecture caused by the mere presence or absence of a sole methyl group at the furanoside 3´-OH function.

Similar structural changes of the *loop-helix motif* combined with a reduction of the *a*-axis and β-angle had been observed beforehand, namely in the crystal structure of TGT co-crystallized with inhibitor **4** (Fig 2). This ligand had been designed to launch a rigid 3-pyridyl-ethynyl needle in the homodimer interface with the intention to destabilize it [22]. Remarkably, the named conformational changes of TGT as well as changes in unit cell parameters did not appear when **4** was soaked into a pre-existing apo-TGT crystal (S2 Fig). In this case, the dimer interface remains nearly unaffected by ligand binding but, instead, ligand **4** with its rigid 3-pyridyl-ethynyl needle adapts its conformation to the preformed protein packing within the crystal. This suggests that conformational changes within the protein as they are likely to occur upon ligand binding in solution may be hampered when ligand molecules are soaked into pre-existing crystals of apo-TGT. Accordingly, binding poses observed in TGT crystals which were soaked with ligands may not necessarily reflect the situation in solution but may suggest artificial, non-relevant binding modes.

This prompted us to reinvestigate a number of related *lin*-benzoguanine-based inhibitors (**5** to **9**; Fig 2) which had been the objects of former studies and, due to their bulky substituents at position 4 of the heterocyclic scaffold (for labeling, see Fig 2), might have potential effects on dimer interface stability as well [19, 20, 26–28]. These compounds had originally been designed with the purpose to inhibit the active site of TGT and, accordingly, had not initially been investigated with respect to any influence on dimer architecture. Since all crystal structures of these ligands in complex with TGT had exclusively been determined by soaking the respective compound into a pre-existing apo-TGT crystal, any such effect most probably would have remained unnoticed (with the exception of ligand **7** that shows an effect on the TGT dimer interface even when soaked into a crystal of apo-TGT; see below). In the present study, we therefore prepared co-crystal structures of TGT with **5** to **9**, which were expected to uncover the influence of these ligands on dimer interface architecture, if there was any.

Indeed, our study revealed further ligands exhibiting significant influence on dimer stability and architecture as transparent in the crystal structures. Mass spectrometry and ITC measurements suggest that our crystallographic findings also mirror the structural properties of the complexes in solution. The systematic comparison of co-crystallized and soaked TGT-ligand complexes provided further examples illustrating that adopted binding modes may depend on the applied crystallization protocol. Moreover, a totally unexpected effect was observed in the co-crystal structure of TGT in complex with **9**. Here, co-crystallization led to a novel crystal packing with a TGT dimer structure showing a monomer arrangement entirely different from that previously reported for bacterial TGTs. The formed *twisted dimer* provides the first example where ligand binding obviously induces a pronounced reorganization of dimer assembly. The impact of this surprising finding on structure-based drug design is discussed in detail.

## Results and discussion

### Overview and intention of crystal structures

To gain insight into potential effects of **5** to **9** on TGT dimer formation and stability, we co-crystallized these ligands with *Z*. *mobilis* TGT and determined the crystal structures of the resulting complexes (TGT∙**5-9**_co_). So far, all available structures of these complexes had been obtained by soaking the ligands into pre-existing apo-TGT crystals (TGT∙**5**-**9**_soak_). In addition, we determined the structure of TGT in complex with **3** after soaking it into a crystal of apo-TGT (TGT∙**3**_soak_), since our initial structure of **3** bound to the enzyme had been gained *via* co-crystallization (TGT∙**3**_co_). Fig 2 lists resolution, space group and unit cell parameters of all crystal structures considered in this study including previously determined ones [19, 23, 27, 29]. Detailed data statistics of the newly determined structures are presented in Table 1.

**Table 1:** Data collection and refinement statistics of X-ray crystal structures.

Complex∙Inhibitor_protocol_	TGT∙3_soak_	TGT∙5_co_	TGT∙6_co_	TGT∙7_co_	TGT∙8_co_	TGT∙9_co_
**(A) Data Collection and Processing**						
collection site	BESSY 14.3	BESSY 14.3	ELETTRA XRD1	BESSY 14.1	BESSY 14.1	DESY P14
no. crystal used	1	1	1	1	1	1
wavelength [Å]	0.89500	0.89500	1.00000	0.91841	0.91841	0.97626
space group	*C*2	*C*2	*C*2	*C*2	*C*2	*P*2_1_
**(B) Diffraction data** ^[a]^						
resolution range [Å]	45.22–1.44 (1.53–1.44)	45.38–1.49 (1.58–1.49)	42.53–1.25 (1.33–1.25)	42.53–1.36 (1.44–1.36)	42.49–1.73 (1.83–1.73)	19.87–1.89 (2.01–1.89)
unique reflections	74015 (11865)	67096 (10734)	105188 (1676)	81972 (13067)	38683 (6142)	67732 (10527)
*R* (*I*)_sym_ [%]^[b]^	5.1 (49.5)	6.1 (48.0)	5.5 (49.1)	3.7 (48.4)	5.6 (49.3)	8.2 (49.3)
completeness [%]	99.5 (99.1)	99.6 (99.0)	99.1 (97.9)	99.1 (97.9)	95.7 (94.9)	98.3 (95.8)
multiplicity	3.4 (3.3)	3.4 (3.3)	3.3 (3.3)	3.8 (3.8)	2.9 (2.8)	4.3 (4.2)
*I*/σ [*I*]	14.9 (2.9)	15.2 (3.1)	11.3 (2.1)	17.7 (2.6)	14.5 (2.2)	11.3 (2.7)
Matthews coefficient [Å^3^∙Da^-1^] ^[c]^	2.4	2.4	2.3	2.3	2.3	2.6
Solvent content [%]	49.2	49.3	45.7	45.9	45.8	51.8
Molecules in asymmetric unit	1	1	1	1	1	2
**(C) Refinement with Phenix** [41]						
resolution range [Å]	45.22–1.44	43.38–1.49	42.15–1.25	42.33–1.36	43.49–1.73	19.87–1.89
reflections used in refinement	73978	67060	105178	81969	38682	67714
final *R* values						
*R*_free_ [%]^[d]^	16.6	15.6	15.8	17.3	20.2	22.0
*R*_work_ [%]^[e]^	13.3	12.5	13.7	14.3	18.1	19.1
no. of atoms (non-hydrogen)						
protein atoms	3024	3009	2866	2835	2793	A: 2864 B: 2852
water molecules	396	408	324	275	223	434
inhibitor atoms	26	26	27	25	24	46 (2 x 23)
RMSD from ideality						
Bond angles[°]	0.8	0.8	0.8	0.8	0.8	0.9
Bond lengths [Å]	0.005	0.005	0.005	0.005	0.006	0.008
Ramachandran plot^[f]^						
most favored regions [%]	95.5	94.0	93.7	94.6	93.7	93.7
additionally allowed regions [%]	4.2	5.7	5.9	5.0	5.9	6.0
generously allowed regions [%]	0.3	0.3	0.3	0.3	0.3	0.3
Mean *B-*factor [Å^2^] ^[g]^						
protein atoms	18.0	16.6	17.8	19.6	23.6	A: 25.6 B: 27.3
water molecules	35.3	33.7	35.4	35.8	34.9	34.6
inhibitor atoms	17.4	11.9	12.8	16.8	17.6	A: 17.0 B: 20.2

^[a]^Values in parentheses are statistics for the highest resolution shell.

^[b]^*R*(*I*)_sym_ = [∑_h_∑_i_|*I*_i_(h)-‹*I*(h)›|/∑_h_∑_i_*I*_i_(h)] ∙ 100, where ‹*I*(h)› is the mean of the *I*(h) observation of reflection h.

^[c]^Calculated with MATTPROB [38, 39],

^[d]^*R*_free_ was calculated as shown for *R*_work_ but on refinement-excluded 5% of data.

^[e]^*R*_work_ = ∑_hkl_ |*F*_o_−*F*_c_| / ∑_hkl_ |*F*_o_|.

^[f]^ Determined with PROCHECK [60].

^[g]^ Calculated with MOLEMAN [61].

All examined ligands are based on the parent *lin*-benzoguanine scaffold, which binds similarly to the enzyme in all structures. It is accommodated by the guanine-34/preQ_1_ binding site where it is sandwiched between the side chains of Tyr106 and Met260 and forms a total of seven hydrogen bonds (H-bonds) to Asp102, Asp156, Gln203, Gly230 and Leu231 (Fig 1) [26, 30]. In **1** to **6**, the *lin*-benzoguanine contains a 2-methylamino substituent, which allows for a further H-bond to the main-chain carbonyl of Ala232. For the present study, however, this feature is of minor relevance.

### Methylation of the furanoside 3´-hydroxyl group clearly impacts the architecture of the TGT dimer interface

The co-crystal structures of **2** and **3** (TGT∙**2**_co_ and TGT∙**3**_co_) had been published by us previously presenting a detailed analysis of the adopted binding poses [23]. Both compounds bear a furanoside substituent at position 4, which solely differs in the absence (**2**) or presence (**3**) of a methyl group attached to the 3´-OH group of the furanoside (Fig 2). The 4-substituents of both ligands occupy the R34/P35-pocket with the furanosides mimicking the 5´-phosphate group of nucleotide-35 of a bound tRNA substrate. Thereby, however, they adopt considerably divergent conformations (Fig 3a).

In TGT∙**2**_co_, the 3´-OH group of the furanoside, which is methylated in **3**, donates an H-bond to the carboxylate group of Asp280. In addition, compared to apo-TGT (PDB entry: 1P0D [18]; S1a Fig), several structural changes are observed in the vicinity of the bound furanoside, which are exhaustively described in reference [23] but of minor relevance for the present study (for details see S1c Fig). Importantly, the positions and conformations of residues Val45, Leu68 and Val282, forming the *hydrophobic floor* of the R34/P35-pocket, as well as the *loop-helix motif* (loop *β*1*α*1 plus helix *α*1 = residues 47 to 55 plus 56 to 62) are not influenced by the binding of **2**.

Since in ligand **3** the furanosyl 3´-OH group is methylated, its H-bond donor functionality is lost and no contact to Asp280 is formed in TGT∙**3**_co_ (Fig 3a and S3b Fig). The additional methyl group of **3** induces a strikingly different conformation of the 4-substituent compared with that of **2** in TGT∙**2**_co_, in which its furanosyl moiety pushes Asn70 toward His73. Remarkably, compared to the crystal structure of apo-TGT, a similar movement of Asn70 (entailing a visible shift of two residues preceding and two residues succeeding Asn70) is present in all considered following crystal structures unless stated otherwise. Furthermore, the furanosyl moiety in TGT∙**3**_co_ bears against the *hydrophobic floor* of the R34/P35-pocket thereby forcing Leu68 to move toward Val45, which, consequently, is also displaced. Strikingly, the shift of Val45 entails the dislocation of Gly46 and Thr47 by as much as 6.6 Å (Thr47 C*α*).

The large movement of Thr47 has strong influence on the geometry of the *loop-helix motif* usually shielding a cluster of aromatic residues within the TGT homodimer interface from water access. While the three N-terminal residues of the loop *β*1*α*1 (Thr47 to Ala49) now follow a completely altered course, the remaining part of this loop (residues 50 to 55) as well as the helix *α*1 (residues 56 to 61) are no longer crystallographically resolved in TGT∙**3**_co_. Within the dimer interface, the *loop-helix motif* contributes a considerable number of interactions between both subunits (six H-bonds, six salt bridges and 70 van-der-Waals contacts; based on PDB entry 1P0D [18]). Thus, its collapse, as induced by **3**, will supposedly cause a severe destabilization of TGT dimer interface contacts. Notably, the breakdown of the *loop-helix motif* is concomitant with significant changes of unit cell parameters. While the *a*-axis is reduced by about 6 Å, the β-angle shrinks by more than 2° compared to common *Z*. *mobilis* TGT crystals.

### The influence of ligand 3 on dimer architecture is not revealed by soaking 3 into apo-TGT crystals

In a former study, we had shown that the structure of TGT co-crystallized with **4** (TGT∙**4**_co_) reveals a binding mode which is significantly different from that seen in the structure obtained after soaking **4** into an apo-TGT crystal (TGT∙**4**_soak_) (Fig 2 and S2 Fig) [22]. Similar to TGT∙**3**_co_, the adopted binding mode in TGT∙**4**_co_ leads to a pronounced rearrangement of the *loop-helix motif* accompanied by conspicuous changes of unit cell parameters (reduction of the *a*-axis and shrinking of the β-angle). In contrast, neither any change in the geometry of the *loop-helix motif* nor in unit cell parameters is seen in TGT∙**4**_soak_. This prompted us to investigate whether the binding mode of **3** also depends on the applied co-crystallization or soaking protocol. Therefore, we determined a structure obtained from a crystal of apo-TGT soaked with this ligand (TGT∙**3**_soak_).

Indeed, the unit cell parameters in TGT∙**3**_soak_ are virtually identical to those usually present in *Z*. *mobilis* TGT crystals. Yet, compared to TGT∙**3**_co_, significant differences in the binding mode of the ligand´s 4-substituent are observed (Fig 3b). While clear electron density is visible for the entire inhibitor in TGT∙**3**_co_, its furanosyl moiety is poorly defined in the electron density of TGT∙**3**_soak_. In fact, the occupancy of the furanosyl ring in TGT∙**3**_soak_ was refined to 57% only and, as it proved impossible to reliably spot the 2-methoxy and the 3-methyl group of this moiety, they are missing in the final model. In addition, while two water molecules interact with the furanosyl moiety in TGT∙**3**_co_, no electron density for any water molecule is present at equivalent positions in TGT∙**3**_soak_. This suggests that the 4-substituent in TGT∙**3**_soak_ is considerably more flexible or scattered over multiple arrangements compared to TGT∙**3**_co_. Furthermore, in TGT∙**3**_soak_ the center of the furanosyl ring is shifted by about 1.3 Å toward the solvent (Fig 3b and S3a and S3b Fig).

Aside from missing electron density for the Gln107 side chain in TGT∙**3**_soak_, all residues lining the guanine-34/preQ_1_ binding site including the catalytic residues Asp102 and Asp280 superimpose perfectly well in TGT∙**3**_soak_ and TGT∙**3**_co_. Likewise, in both structures a water molecule bridges *N*3 of the *lin*-benzoguanine scaffold and the endocyclic oxygen of the furanosyl ring (S3a and S3b Fig). Significant differences, however, are observed in the vicinity of the bound furanosyl ring. While the stretch of residues comprising Leu68 to His73 follows a clearly defined course in TGT∙**3**_co_, the same stretch adopts two alternative conformations (A and B) in TGT∙**3**_soak_ (Fig 3b). Conformation B is virtually identical to that seen in the structure with the 4-unsubstituted ligand **1** (S1b and S3a Figs). Yet, as the side chains of Leu68 and Asn70 form unacceptably close contacts to the ligand´s furanosyl, this conformation is not compatible with the present furanosyl placement. Consequently, it was refined to an occupancy of 43%, which is complementary to the occupancy of the furanosyl moiety. In contrast, conformation A allows binding of the ligand´s furanosyl portion as displayed in the crystal structure and was refined to an occupancy of 57%. The course of Leu68 to His73 in conformation A is highly similar to that in TGT∙**3**_co_ except for no electron density being present for the side chain of His73 and a different conformation adopted by the side chain of Leu68 (Fig 3b). The latter also differs from that observed in apo-TGT and constitutes the only change in the *hydrophobic floor* induced by the ligand in TGT∙**3**_soak_. Importantly, in contrast to TGT∙**3**_co_, the position of Val45 remains unchanged. Consequently, the concomitant large movement of Thr47, which leads to the collapse of the *loop-helix* motif in TGT∙**3**_co_, is not observed in TGT∙**3**_soak_. Although no proper electron density is present for Thr47, it likely remains close to its original position, leaving the *loop-helix motif* and, thus, the dimer interface unaffected.

In summary, the binding of ligand **3** to TGT in solution most likely induces pronounced conformational adaptations of TGT allowing energetically favorable interactions between the ligand´s furanosyl and the protein as observed in TGT∙**3**_co_. Once, however, protein flexibility is constrained by the crystal packing of a pre-formed apo-crystal the mutual induced-fit adaptions of protein and ligand appear strongly reduced in TGT∙**3**_soak_ and favorable interactions between the ligand´s furanosyl and the R34/P35-pocket are hampered. Supposedly, to a certain extent, the protein largely retains its original conformation and the ligand´s furanosyl adopts no well-defined conformation, thus remaining invisible in the crystal structure. No electron density is detectable in TGT∙**3**_soak_ for this moiety, which would be compatible with conformation B of the Leu68-to-His73 stretch. To some extent, however, partial adaptation of the Leu68-to-His73 stretch to the ligand may occur (course A of this stretch in TGT∙**3**_co_) facilitating interactions between the protein and the ligand´s furanosyl. Yet, the conformational changes within the protein, observed in TGT∙**3**_soak_, remain locally confined. Consequently, this structure does not allow assessing the situation in solution, not to mention the ligand´s impact on dimer stability. In the course of the present study, we made similar observations for a number of further ligands, which are described in the following.

### Ligands 5 and 6, differing in aliphatic ring size, exert differing influence on TGT dimer architecture

A structure of **5** in complex with TGT, obtained by soaking the single-digit nanomolar inhibitor into preformed TGT crystals (TGT∙**5**_soak_), was published previously by us [27]. The 4-(cyclopentyl-methylamino)ethyl substituent is well-defined in the electron density and deeply penetrates into the R34/P35-pocket (Fig 3c and S3c Fig). This placement allows for energetically favorable interactions between the *hydrophobic floor* and the ligand´s cyclopentyl moiety, which adopts a twist envelope conformation. Compared to apo-TGT, the cyclopentyl moiety causes a slight rotation of the Leu68 side chain (compare Fig 3c and S1a Fig). However, this rotation has no influence on the position of Val45 or any further nearby residue. Close to the guanine-34/preQ_1_ binding site, a water molecule mediates an interaction between *N*3 of the *lin*-benzoguanine scaffold and the 4-substituent´s secondary amino group. The latter is likely protonated enabling the formation of a salt bridge with the carboxylate group of Asp280.

The newly determined co-crystal structure TGT∙**5**_co_ displays a binding mode closely resembling that in TGT∙**5**_soak_. Only minor differences are found for a small number of residues lining the R34/P35-pocket (S3d Fig). These do not have major impact on the geometry of the protein and leave the *loop-helix motif* within the dimer interface virtually unaffected (Fig 3c and S3c and S3d Fig).

Interestingly, in TGT∙**5**_co_ a chloride ion, confirmed by its anomalous scattering contribution (see Materials and methods), is coordinated by the carboxamide of Gln107 and the backbone amides of Thr71 and Tyr72 (S5 Fig). Presumably, it is also present in TGT∙**4**_co_, but was erroneously refined as a water molecule leading to an exceedingly low *B*-factor of 4.7 Å^2^.

Similar to **5**, ligand **6** constitutes a potent inhibitor of bacterial TGT with single-digit nanomolar affinity. Both inhibitors only differ by the replacement of a terminal cyclopentyl (**5**) by a cyclohexyl ring (**6**) in the 4-substituent. Like TGT∙**5**_soak_, the crystal structure TGT∙**6**_soak_ was published by us previously [27]. Similar to TGT∙**5**_soak_ and TGT∙**5**_co_, a water molecule bridges *N*3 of the *lin*-benzoguanine and the 4-substituent´s secondary amino group in TGT∙**6**_soak_, which forms a salt bridge to the carboxylate group of Asp280 (Fig 3c and 3d and S3c–S3e Fig). The entire 4-substituent of **6** is well-defined in the electron density with its cyclohexyl moiety adopting a chair conformation. It deeply penetrates into the R34/P35-pocket making extensive van-der-Waals interactions with the *hydrophobic floor* of this pocket. The aliphatic ring of **6** slightly shifts the side chain of Leu68, yet, without affecting the position of Val45 or any further nearby residues. Accordingly, as in TGT∙**5**_soak_ and TGT∙**5**_co_, no impact on the geometry of the *loop-helix motif* and the dimer interface architecture is experienced.

In contrast to TGT∙**5** structures, TGT∙**6**_co_ is strongly altered in comparison to TGT∙**6**_soak_. In TGT∙**6**_co_, the ligand´s cyclohexyl moiety penetrates approximately 0.6 Å more deeply into the R34/P35-pocket (Fig 3d and S3e and S3f Fig). Thereby it comes unfavorably close to Val45 (2.7 Å), which, consequently, is displaced from its original position by about 2 Å (C*α*). This entails a pronounced shift of adjacent Gly46 and Thr47 residues, whose original positions are then occupied by three and two water molecules, respectively. As a result, the first three residues of the *loop-helix motif*, Thr47 to Ala49, take an entirely different course and the following residues, Thr50 to Thr62, are, due to disorder, no longer crystallographically resolved. As in TGT∙**3**_co_ and TGT∙**4**_co_, these extensive structural rearrangements are concomitant with a reduction of the unit cell *a*-axis and β-angle. Accordingly, while ligand **5** exerts no influence on dimer architecture in the co-crystallized complexes, the closely related ligand **6**, whose aliphatic ring system is expanded by a single methylene group, is able to cause the breakdown of the *loop-helix motif*, which may entail a significant destabilization of the dimer interface.

As in TGT∙**5**_co_, a chloride ion bound to Gln107, Thr71 and Tyr72 was identified in TGT∙**6**_co_ (S5 Fig). Most probably, it is also present in TGT∙**6**_soak_ but there it was refined as a water molecule leading to the low *B*-factor of 8.2 Å^2^.

### Co-crystallization of TGT with 7 reverses the space group change observed in soaked crystal structure

TGT∙**7**_soak_ was determined by us in 2007 [19]. It reveals a unique reduction of crystal symmetry from space group *C*2 with one TGT monomer per asymmetric unit (resulting in two identical dimers per unit cell) to space group *P*2. In *P*2 the asymmetric unit consists of two conformationally non-identical monomers, referred to as TGT_1_∙**7**_soak_ (chain A) and TGT_2_∙**7**_soak_ (chain D).

TGT_1_∙**7**_soak_ exhibits typical features commonly found in structures of TGT in complex with 4-substituted *lin*-benzoguanines obtained by soaking. In this structure, the 2-(4-methoxyphenyl)ethyl substituent seems rather flexible as indicated by moderately defined electron density [19]. Compared to the apo-TGT structure, only minor ligand-induced conformational changes in the R34/P35-pocket are observed. Thus, the side chains of Leu68 and Thr47 are rotated to avoid clashes with the terminal methoxy group of the 4-substituent (compare S1a and S4a Figs). Yet, overall no significant influences on the geometry of adjacent structural elements within the protein are detected (Fig 3e).

In contrast, the situation in TGT_2_∙**7**_soak_ is strongly reminiscent of what is observed in many co-crystal structures of TGT with 4-substituted *lin*-benzoguanines. Here, the entire ligand is excellently defined in the electron density concomitant with average *B*-factors of the ligand (30.7 Å^2^), particularly of the 4-substituent (32.9 Å^2^), which are clearly lower than in TGT_1_∙**7**_soak_ (45.0 Å^2^ / 53.4 Å^2^) [19]. The *p*-methoxyphenyl moiety immerges, compared to TGT_1_∙**7**_soak_, significantly deeper into the R34/P35-pocket. At the *hydrophobic floor* of this pocket, it comes unacceptably close to Leu68 (1.0 Å), which is shifted toward Val45 (Fig 3e and S4b Fig). Consequently, Val45 is displaced and the neighboring residues, Gly46 and Thr47, are considerably dislocated. In addition, a collapse of the *loop-helix motif* from Ala48 to Thr62 is caused and thus, these residues remain crystallographically invisible in TGT_2_∙**7**_soak_.

Co-crystallization of TGT with **7** was done in this study leading to TGT∙**7**_co_, in which the original *C*2 space group was re-established although, similar to TGT∙**3**_co_, TGT∙**4**_co_ and TGT∙**6**_co_, the *a*-axis and the β-angle are clearly reduced (Fig 2). In TGT∙**7**_co_, the ligand adopts a virtually identical conformation to TGT_2_∙**7**_soak_ (Fig 3e and S4b Fig). Within the *hydrophobic floor*, the terminal methoxy group of the ligand pushes Leu68 toward Val45, which is dislocated from its original position. Val282 as well as the neighboring Cys281 become disordered as indicated by ill-defined electron density. The shift of Val45 involves a pronounced movement of Gly46 and Thr47, which causes the collapse of the *loop-helix motif*. While Ala53 to Thr62 are not visible in the electron density, the remaining part of this motif follows a completely different course compared to what is usually observed.

### Shrinking OMe to Me at the 4-substituent still disturbs TGT dimer interface architecture

The 4-substituent of **8**, a 2-(*p*-toluyl)ethyl moiety, is highly similar to that of **7** and the corresponding TGT∙**8**_soak_ was reported by us previously [19]. Here, reminiscent of the situation in TGT∙**3**_soak_, the Gly69 to His73 segment adopts two different conformations (A and B), which refine to an occupancy of 49% and 51%, respectively (Fig 3f and S4c Fig). Conformation A is nearly indistinguishable from that in TGT∙**1**_soak_. In contrast, conformation B leads to an expansion of the R34/P35-pocket with Asn70 shifted off its original position. Here, the course of the Gly69-to-His73 stretch corresponds to that found for most of the TGT complexes discussed in this paper (except TGT∙**2**_co_). By analogy with TGT∙**3**_soak_, the 4-substituent of **8** is partially ill-defined in the electron density as reflected by conspicuously high *B*-factors assigned to its atoms [19]. However, in contrast to TGT∙**3**_soak_, the 4-substituent in TGT∙**8**_soak_ is definitely compatible with course A of the Gly69-to-His73 segment (resembling the conformation of this segment in TGT∙**1**_soak_). Yet, although the occupancy of the 2-(*p*-toluyl)ethyl moiety was set to 100%, the electron density map of TGT∙**8**_soak_ shows further, rudimentary density indicating an alternative conformation of this moiety. Obviously there, the 4-substituent penetrates considerably more deeply into the R34/P35-pocket thereby forcing the Gly69-to-His73 segment into conformation B. Compared to apo-TGT, the side chain of Val282 is rotated by about 70° in TGT∙**8**_soak_ (compare S1a and S4c Figs). However, no conformational changes are observed for Leu68 and Val45 within the *hydrophobic floor* and C*α* of Thr47 retains its original position. Thus, the *loop-helix motif* remains intact in TGT∙**8**_soak_.

In TGT∙**8**_co_ determined in this study, the 2-(*p*-toluyl)ethyl moiety is present in a single conformation (Fig 3f and S4d Fig), which corresponds to the above-mentioned rudimentary electron density observed in TGT∙**8**_soak_. Additionally, the course of the Gly69-to-His73 segment in TGT∙**8**_co_ is highly similar to conformation B of this stretch in TGT∙**8**_soak_. The toluyl moiety of the ligand deeply immerges into the R34/P35-pocket thereby coming unfavorably close to Leu68 within the *hydrophobic floor* (2.6 Å). Consequently, Leu68 is shifted toward Val45 and displaces the latter from its original position. As repeatedly observed, the shift of Val45 provokes a pronounced movement of Gly46 and Thr47, which ultimately rearranges the *loop-helix motif*. Despite the fact that the crystallographically undefined section of this motif in TGT∙**8**_co_ is relatively short (Lys55 to Arg60), it takes a completely different course in comparison to what is normally seen in crystal structures of *Z*. *mobilis* TGT. Four well-defined water molecules are identified in close vicinity of Thr47 indicating that its original task, to shield the dimer interface from water access, is no longer performed by the former *loop-helix motif* in the geometry found in TGT∙**8**_co_ (Fig 3f and S6 Fig). Here again, the restructuring and partial collapse of the *loop-helix motif* is accompanied by a reduction of the *a*-axis and β-angle (Fig 2).

### Removal of the terminal methyl group in 9 triggers a profound rearrangement of the homodimer

**Fig 4:**
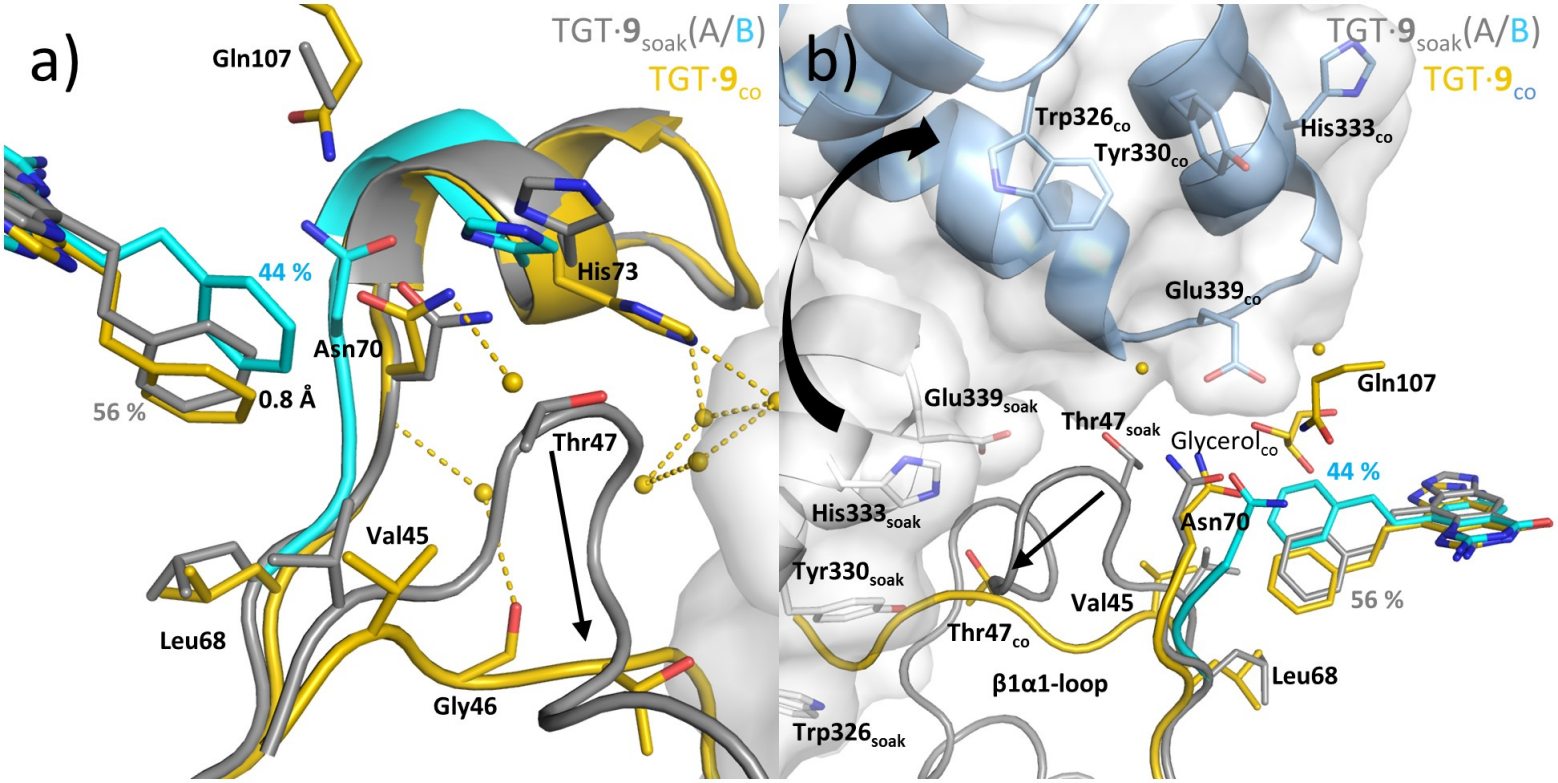
Binding modes and structural rearrangements of 9. **a)** TGT∙**9**_soak_ (conformation A gray and conformation B cyan) and TGT∙**9**_co_ (yellow), **b)** View from an alternative direction including the second monomer of TGT∙**9**_co_ in light blue and with gray solvent accessible surface. Black arrows indicate positional differences between the crystal structures of different crystallization protocols. Inhibitor **9** displaces the *β*1*α*1-loop in the co-crystal structure and triggers the formation of a new *twisted dimer* (see Figure 4d). Yellow dashed lines indicate H-bonds from selected water molecules (2.7–3.4 Å).

Ligand **9** solely differs from **8** by the removal of the terminal toluyl methyl group. In TGT∙**9**_soak_, previously determined by us [19], the 4-substituent adopts two conformations (A and B) which can be interconverted by rotating about the covalent bond attaching the 4-substituent to the *lin*-benzoguanine scaffold (Fig 4 and S4e Fig). In conformation B (occupancy of 44%), the substituent remains more solvent exposed, while it deeply penetrates into the R34/P35-pocket in conformation A (occupancy of 56%). Reminiscent of TGT∙**3**_soak_ and TGT∙**8**_soak_, the two conformations of the ligand´s 4-substituent are accompanied by two conformations (A and B) of the TGT segment comprising Leu68 to His73. Also in TGT∙**9**_soak_, conformation B of this stretch is highly similar to that observed in TGT∙**1**_soak_. Conformation A leads to an enlargement of the R34/P35-pocket with C*α* of Asn70 being shifted by 2.3 Å. Within the *hydrophobic floor* the side chain of Leu68 is rotated by about 90°, which, however, has no influence on Val45. Accordingly, the positions of Thr47, the *loop-helix motif* and the architecture of the dimer interface remain unaltered in TGT∙**9**_soak_.

In this study, we determined TGT∙**9**_co_ revealing a totally unexpected profound influence of ligand **9** on the TGT dimer architecture. The ligand´s 4-substituent adopts only one conformation, resembling conformation A in TGT∙**9**_soak_, although its phenyl moiety penetrates about 0.8 Å more deeply into the R34/P35-pocket (Fig 4 and S4f Fig). In line with a single conformation of the 2-phenylethyl moiety, the Leu68-to-His73 stretch is also observed in only one conformation, which largely corresponds to conformation A of this segment in TGT∙**9**_soak_. By immerging more deeply into the R34/P35-pocket, the phenyl ring of **9** bears against the *hydrophobic floor* and moves Leu68 away from its original position toward Val45. As a result, Val45 is displaced inducing a pronounced shift of the successive residues, Gly46 and Thr47. Similar to TGT∙**3**_co_, TGT∙**4**_co_, TGT∙**6**_co_, TGT_2_∙**7**_soak_, TGT∙**7**_co_ and TGT∙**8**_co_, this leads to a reorganization of the *loop-helix motif*. Thereby, residues 51 to 58 of this motif become disordered as no electron density is observed for these amino acids. In stark contrast to all other mentioned crystal structures, the four N-terminal residues of loop *β*1*α*1, Thr47 to Thr50, take a course, which directly leads into an area within the dimer interface normally harboring a cluster of four aromatic residues. This cluster, consisting of Phe92 as well as of Trp326´, Tyr330´ and His333´ of the dimer mate (Figs 4b and 7a), could be characterized by us as a hot spot, strongly contributing to the stability of the TGT dimer interface [24, 25].

**Fig 5:**
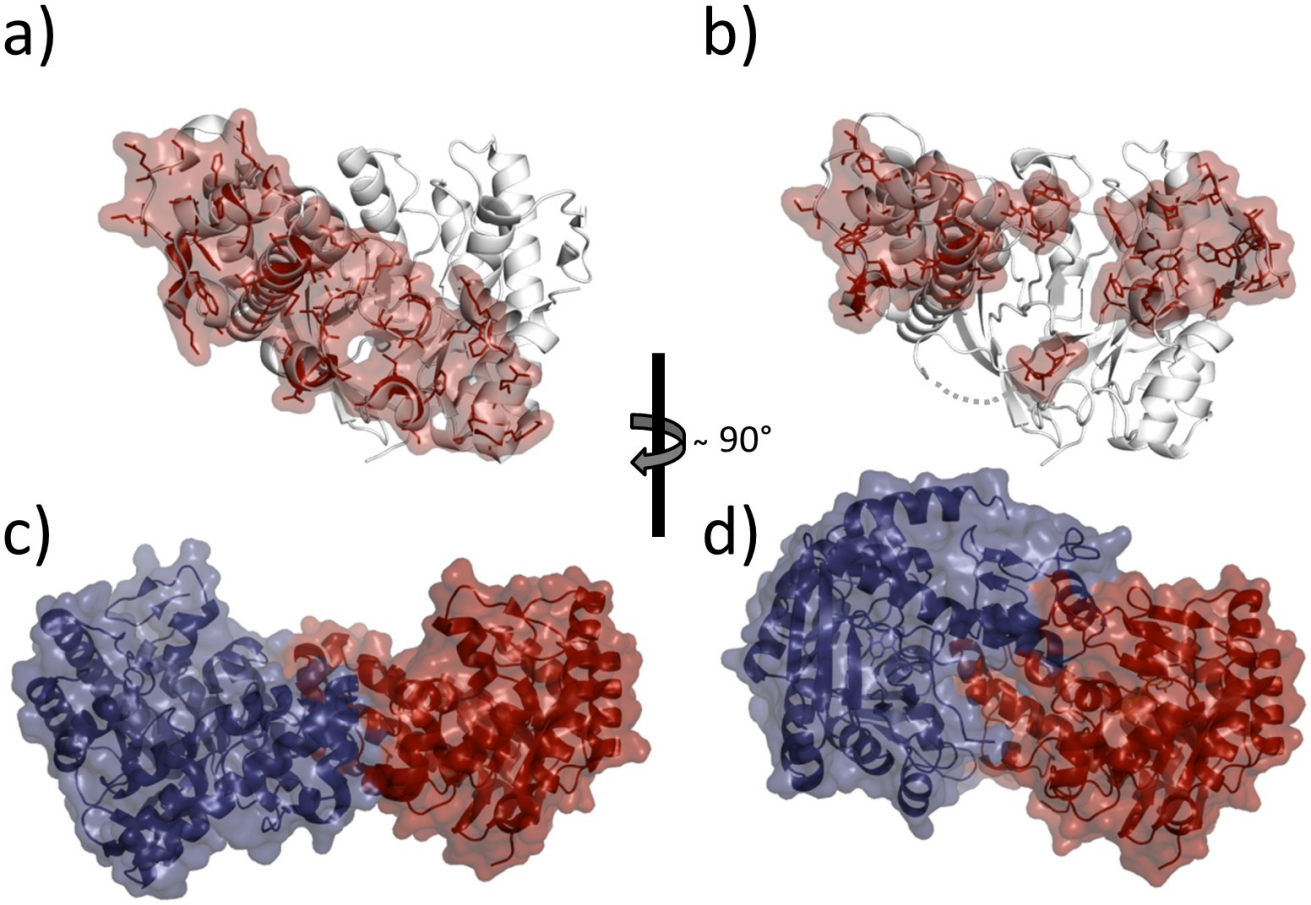
Comparison of alternative TGT dimer interfaces and oligomeric states. Dimer interface areas calculated using PDBePISA49 are highlighted by red surface patches and stick representation. **a)** apo-TGT dimer interface area of monomer 1: 1619 Å^2^ (PDB entry: 1P0D [18]). **b)** twisted dimer interface of monomer 1: 1644 Å^2^ (found in TGT∙**9**_co_) **c)** oligomeric state of apo-TGT dimer (monomer 1 red, monomer 2 blue) **d)** oligomeric state of twisted dimer (monomer 1 red, monomer 2 blue).

**Fig 6:**
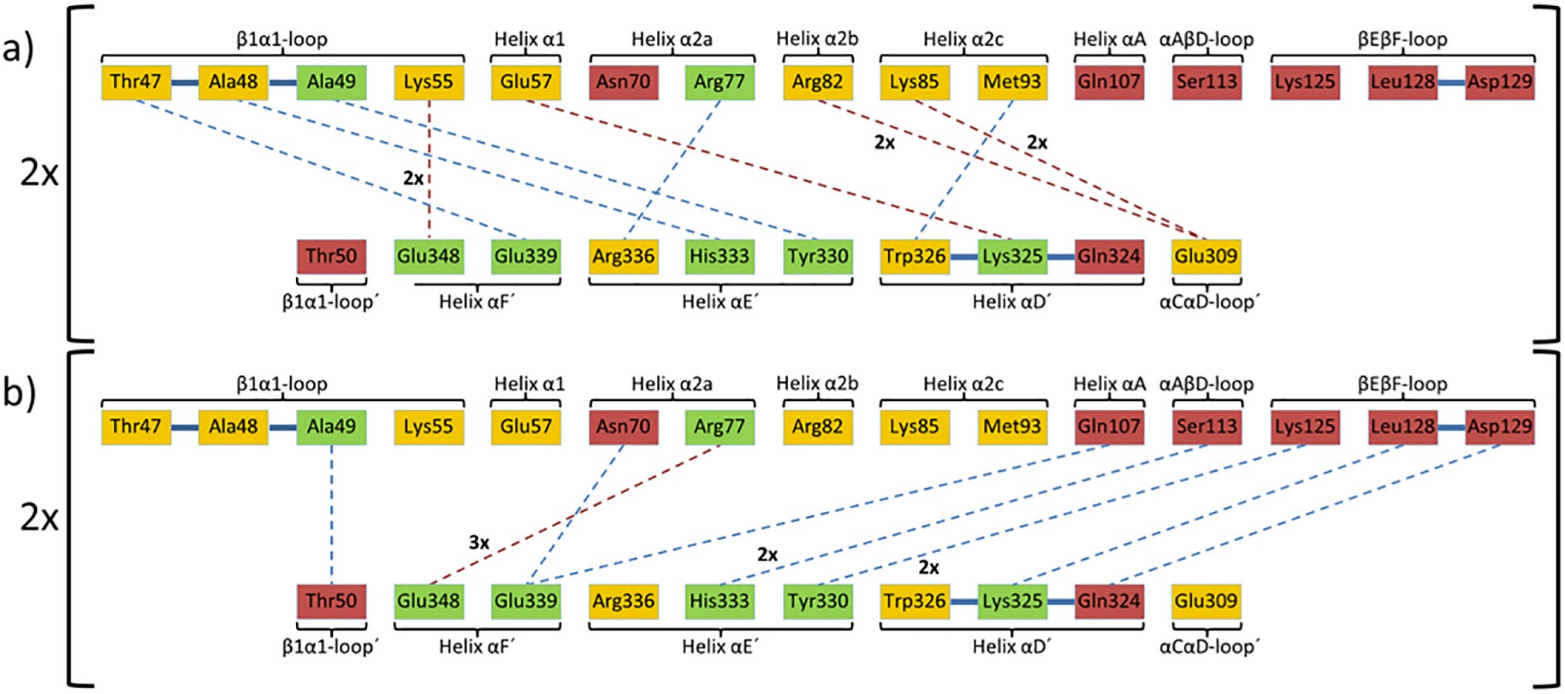
Polar interactions formed in a) apo-TGT (PDB entry: 1P0D [18]) and b) *twisted dimer* found in TGT∙9_co_. Residues that form H-bonds or salt bridges across the interface only in apo-TGT (yellow), in both structures (green), and in the *twisted dimer* (red) are indicated. Contacts were determined by CONTACTSYM [47, 48]. Due to interface symmetry, all displayed interactions occur twice but are depicted only once for the sake of clarity. Only H-bonds (blue dashed lines) and salt bridges (red dashed lines) with distances between 2.6 and 3.7 Å are depicted.

**Fig 7:**
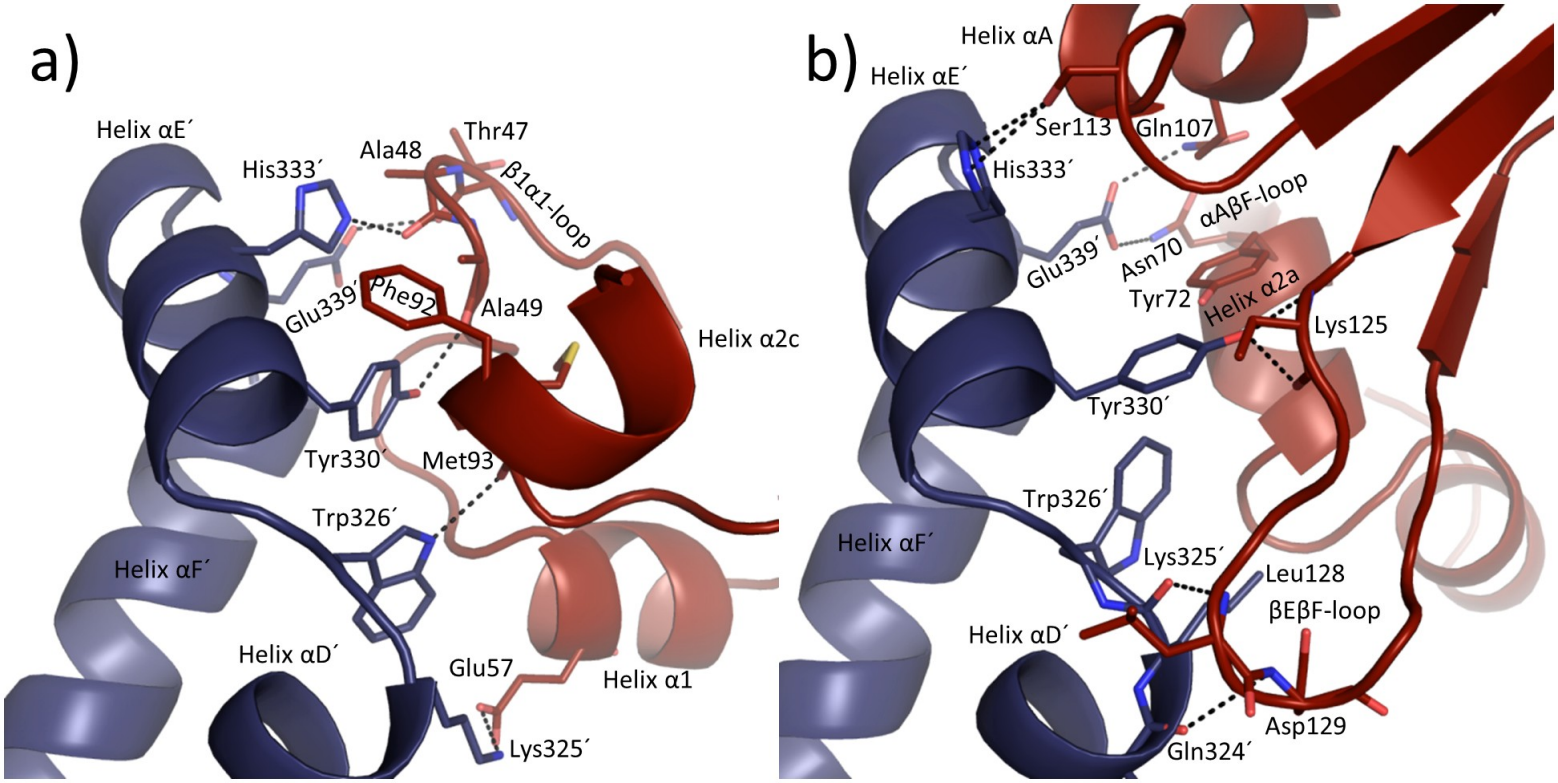
Illustration of the aromatic hot spot regions. H-bonds and salt bridges are displayed as black dashed lines. **a)** Arrangement of the aromatic hot spot region formed in apo-TGT (PDB entry: 1P0D[18]). The *β*1*α*1-loop of the *loop-helix motif* shields this region from water access and can be perturbed by inhibitor binding (monomer 1 red, monomer 2 blue, space group *C*2). **b)** New arrangement of the hot spot region in *the twisted dimer* found in TGT∙**9**_co_ (monomer 1 red, monomer 2 blue, space group *P*2_1_).

Due to the conformation adopted by the Thr47-to-Thr50 stretch in TGT∙**9**_co_, the conventional dimer architecture is not able to persist any more. Obviously, to avoid steric clashes between this stretch and residues Trp326´, Tyr330´ and His333´, the two subunits assemble in a way strikingly different from what is usually observed in crystal structures of bacterial TGT (Fig 5). The conventional dimer interface of bacterial TGT consists of 43 residues per monomer, which form, in addition to 188 van-der-Waals interactions, 10 H-bonds and 14 salt bridges (Fig 6a; PDB entry 1P0D [18]). Thereby, the *loop-helix motif* (loop β1*α*1 plus helix *α*1) interacts with helices *α*E´ and *α*D´ of the dimer mate. In addition, interactions between helices *α*2a and *α*E´, helix *α*2b and loop *α*C´*α*D´, as well as helices *α*2c and *α*D´ are observed. By contrast, the novel dimer interface as present in TGT∙**9**_co_ consists of 46 residues forming 16 H-bonds and 6 salt bridges as well as 196 hydrophobic interactions. Thereby both, helices *α*2a and *α*A, interact with helix *α*F´ while loop *α*A*β*D and *β*E*β*F forms interactions with helices *α*D´ and *α*E´ (Figs 6b and 7b). Loop *β*1*α*1 now interacts with loop *β*1*α*1´ from the second monomer. Notably, the contact surface of the novel dimer in TGT∙**9**_co_, henceforth referred to as *twisted dimer*, amounts to 1644 Å^2^ per monomer (Fig 5b), which does virtually not differ from that of the conventional TGT dimer (1619 Å^2^ in the crystal structure of apo-TGT; Fig 5a). This suggests a considerable stability of this alternative dimer form, which, in the presence of **9**, may, in all likelihood, not be triggered by crystal contact formation but *a priori* exists in solution.

Not surprisingly, the radical reorganization of TGT dimer architecture as present in TGT∙**9**_co_ is accompanied by substantial changes in unit cell parameters and symmetry. As mentioned, *Z*. *mobilis* TGT normally crystallizes in space group *C*2 with one monomer per asymmetric unit, resulting in two identical dimers per unit cell. By contrast, the space group of TGT∙**9**_co_ is *P*2_1_, which still contains two homodimers per unit cell. Here, asymmetric units are related by a two-fold screw axis, thus each contains one homodimer made up of two conformationally non-identical monomers. Yet, the differences observed between both monomers of the asymmetric unit are very small and do not affect the binding mode of the ligand, which is virtually identical in both subunits (RMSD_C*α*_ 0.12Å). We would like to emphasize, that in the presence of **9**, *Z*. *mobilis* TGT reproducibly and inevitably will crystallize in space group *P*2_1_, even if seed crystals of space group *C*2 are added to the crystallization droplet.

### Native MS study of in-solution TGT:Ligand mixtures confirms ligand-induced dimer destabilization

In order to gain insights into the dimer disturbing potential seen in the crystal structures with the different ligands and to collect evidence for such a behavior also present in solution, native ESI-MS was performed. This technique enables to monitor the oligomerization state of TGT at equilibrium under non-denaturing conditions, in the absence or presence of saturating ligand concentrations [15, 22, 24, 25]. Previously, we found that relative proportions of TGT monomer were strongly dependent on experimental (protein concentration, batch, conditioning) and instrumental settings (cone voltage, backing pressure). To ensure reliability, these comparative experiments were thus carried out adhering to strictly similar conditions (see Materials and methods).

**Fig 8:**
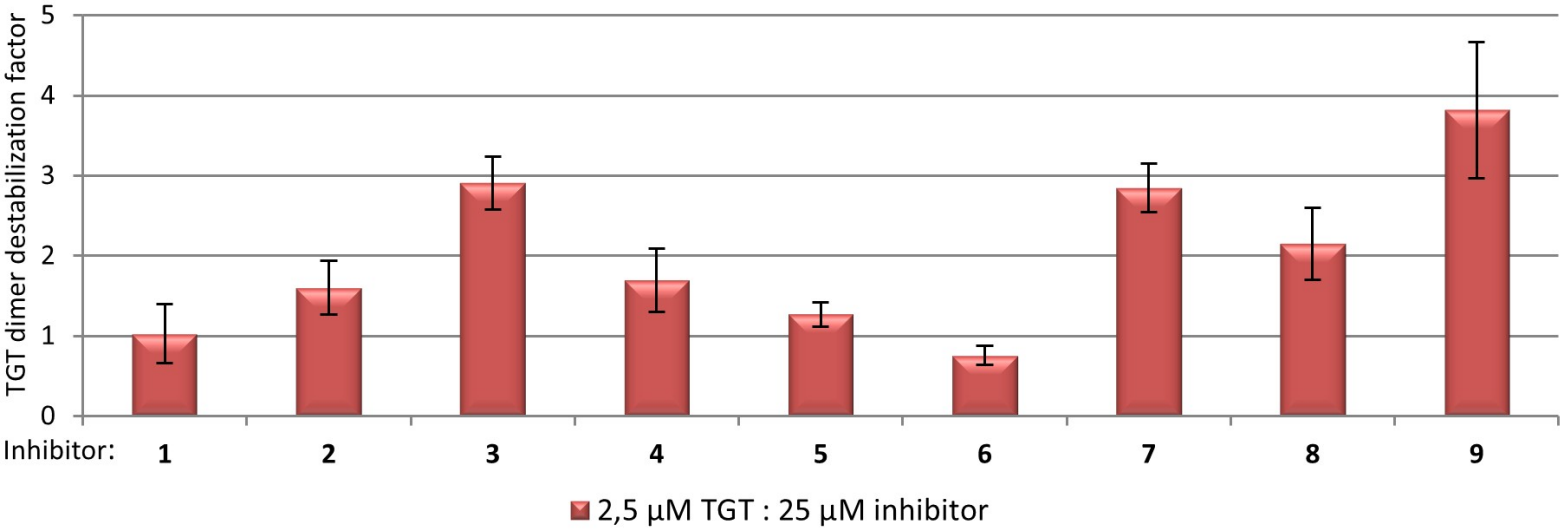
Native MS characterization of TGT dimer disturbing potential from each ligand. Relative quantification of TGT monomers and dimers signals was performed in the absence (TGT 2.5μM) or presence of each ligand (TGT:ligand 1:10 mixture). TGT monomer ratios (%) deduced from each ligand mixture (S7 Fig; S1 Table) were normalized with respect to the apo-condition, enabling to rank inhibitors according to their destabilization factor. Corresponding means and standard deviations (black error bars) are derived from triplicate measurements.

Relative proportions of TGT-monomer signals extracted from individual 1:10 TGT:ligand mixtures (TGT monomer 2.5 μM) were quantified (S7 Fig, S1 Table) and normalized with respect to this value in the absence of any inhibitor. This revealed the dimer destabilization factor (Fig 8). Compared to measurements done in the absence of any ligand, a slightly increased proportion of TGT monomer was observed in the presence of **3**, **7**, **8** and **9**. A small effect might be given for **4** although the effect is at the border of significance (see S1 Table and Immekus *et al*. (2013) [22]). In the corresponding co-crystal structures, all of these ligands were shown to interfere with the geometry of the *loop-helix motif* upon binding to TGT. No effect on dimer stability was observed by native MS for **1**, which lacks any 4-substituent, as well as for **2** and **5**, which, according to TGT∙**2**_co_ and TGT∙**5**_co_, are not able to cause the collapse of the *loop-helix motif*. In addition, ligand **6** did not exert any influence on dimer stability, which is contradictory with the co-crystallized structure, showing that binding of this ligand to TGT impedes the maintenance of the *loop-helix motif*. However, a solubility issue can explain this unexpected finding, since ligand dilution in the buffer (1 M NH_4_Ac pH 7.5) led to apparent precipitation of **6**. To a lower extent, a similar phenomenon was also observed for **4**.

The influence on TGT dimer stability observed for **3**, **7**, **8** and **9** was apparently low in every single case. At least in part, this is clearly due to the non-physiologically high protein concentration used our native MS experiments, which, according to the law of mass action, strongly favors homodimer over monomer formation. Performing these experiments using TGT monomer concentrations of less than 2.5 μM unfortunately led to insufficient signal-over-noise ratios, which stem from technical sensitivity limits and ligand-induced heterogeneity. As the studied ligands differ in affinity, their rates of dissociation from the complexes in the gas phase can slightly deviate with measurement conditions. This effect can take an impact on the comparability of the determined monomer/dimer ratios in the gas phase.

Noteworthy, the most significant increase of normalized monomer proportion was measured for **9** inducing the formation of the *twisted dimer* upon binding to TGT. During TGT crystallization the presence of **9** in saturating concentration exclusively leads to crystals with space group *P*2_1_ (consisting of the *twisted dimer*) even when apo-TGT micro-crystals with space group *C*2 are added. Presumably, in solution this inhibitor allows solely the presence of the *twisted dimer* since even low amounts of the conventional dimer would unavoidably result in the growth of pre-formed *C*2 seed crystals. Although native MS does not allow directly uncovering which dimer form is actually present in solution, the increase of relative monomer proportion caused by **9** suggests a lower stability of the *twisted dimer* in comparison to the conventional one.

### Inhibition constants and thermodynamic profiles

The inhibition constants (*K*_i_) of ligands **1** to **9**, which are listed in Fig 2, were determined previously (except that of **4**, which was determined in this study)[19, 20, 23, 26] *via* a well-established assay monitoring the TGT-catalyzed incorporation of radio-labeled guanine into tRNA. The relatively weak inhibition of TGT by **7** to **9**, exhibiting *K*_i_ values in the single-digit micromolar range, is caused by the fact that, unlike **1** to **6**, these inhibitors lack the methylamino group attached to position 2 of the *lin*-benzoguanine scaffold. This substituent addresses the ribose-33/uracil-33 pocket by forming an H-bond to the main chain carbonyl of Ala232 (Fig 1 and S3 and S4 Figs). Compared to the unsubstituted *lin*-benzoguanine (*K*_i_ = 4.1 ± 1.0 μM [26]) the presence of this 2-substituent increases the affinity of ligand **1** (Fig 2) by a factor of 70 [26].

To further analyze the binding properties of ligands studied in this contribution we decided to establish the thermodynamic analysis of a representative subset *via* isothermal titration calorimetry (ITC) (data for **1** had been determined previously [29]). ITC enables the simultaneous determination of affinity (*K*_d_) and stoichiometry (*n*) as well as enthalpic contribution (Δ*H*^*0*^). The entropic (-*T*Δ*S*^*0*^) term to Gibbs free energy (Δ*G*^*0*^ = Δ*H*^*0*^—*T*Δ*S*^*0*^) results from Δ*G*^*0*^–Δ*H*^*0*^ difference [31, 32]. Due to insufficient availability of some compounds in high purity, quantity and sufficient solubility to perform proper ITC measurements, we decided to focus our investigation on **1**, **2**, **3** and **5**.

**Fig 9:**
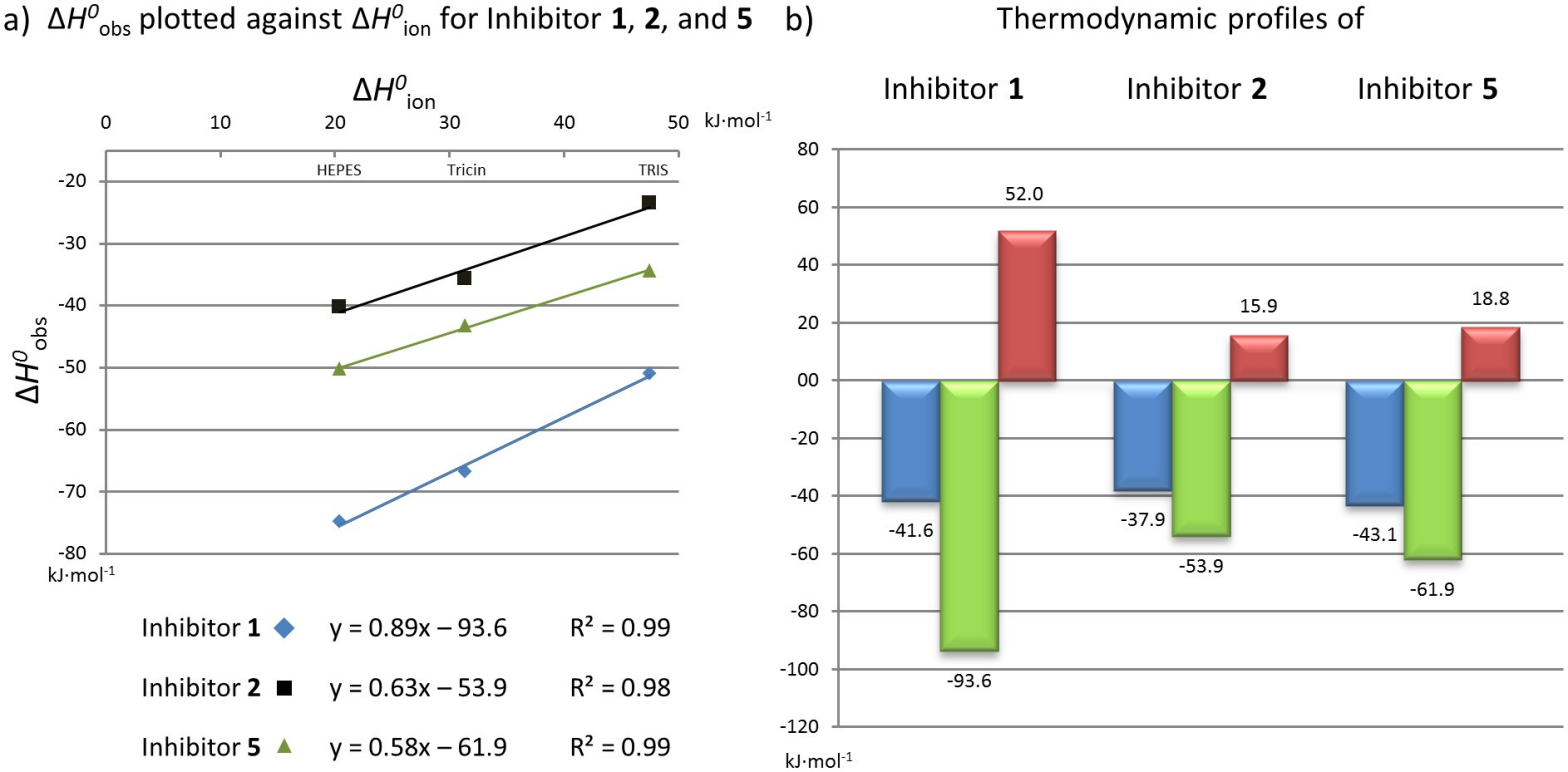
Plot of Δ*H*^*0*^_obs_ against the buffer Δ*H*^*0*^_ion_ and thermodynamic profiles. **a)** Measured heat signal (Δ*H*^*0*^_obs_) is plotted against the ionization enthalpy of applied buffers (HEPES, Tricin, TRIS) to extract the corrected enthalpy of binding (Δ*H*^*0*^_bind_). Inhibitor **1** blue, **2** black, and **5** green. **b)** Thermodynamic parameters Δ*G*^*0*^ (blue) and corrected values for Δ*H*^*0*^_bind_ (green) and -TΔ*S*^*0*^ (red) in kJ∙mol^-1^.

We recently discovered that the binding of *lin*-benzoguanine derivatives is associated with protonation effects that are superimposed to the heat signal of ligand binding to TGT. Systematic analysis of the protonation inventory of the tricyclic scaffold revealed that the source of these overlying effects is the protonation of *N*5 of the *lin*-benzoguanine scaffold [29]. Such protonation effects become transparent when ITC measurements are performed from at least three different buffers (see Materials and methods). The overlaid ionization enthalpy (Δ*H*^*0*^_bind_) of the protonation step was determined as the intersection of the linear regression line obtained by plotting the observed enthalpies of the studied ligands (Δ*H*^*0*^_obs_) against the heat of ionization of the corresponding buffers (Δ*H*^*0*^_ion_) (Fig 9a). Interestingly, for ligand **3**, which possesses a similar affinity to **2** according to our radio-labeled displacement assay, no heat effects at all could be detected in any of the applied buffers. Accordingly, the thermodynamic data obtained from our ITC experiments for **1**, **2** and **5** are listed in Table 2, while representative thermograms are given in S8 Fig. The positive slope of the linear regression reveals the amount of protons, picked up during the binding event. The examined inhibitors **2** and **5**, on average receive 0.61 ± 0.04 protons per mole of the formed complex, which is in reasonable agreement with the results obtained for **1** (0.89) and similar compounds [29].

**Table 2:** Inhibition constants and thermodynamic data at the applied buffers.

Inhibitor	*K*_i_ [nM]	*K*_d_ [nM]	Δ*G*^*0*^ [kJ∙mol^-1^]	Buffer	Δ*H*^*0*^_obs_/Δ*H*^*0*^_bind_ [kJ∙mol^-1^]	-TΔ*S*^*0*^^[a]^ [kJ∙mol^-1^]
**1**	58 ± 36^*26*^	52 ± 7^*29*^	-41.6 ± 0.3^*29*^	HEPES	-74.8 ± 1.8^*29*^	34.2 ± 1.9^*29*^
				Tricin	-66.7 ± 0.1^*29*^	25.1 ± 0.3^*29*^
				TRIS	-50.9 ± 0.9^*29*^	9.3 ± 0.9^*29*^
			**ion. corrected**		**-93.6**	**52.0**
**2**	217 ± 81^*23*^	275 ± 49	-37.9 ± 0.4	HEPES	-40.2 ± 2.2	2.4 ± 2.1
				Tricin	-35.6 ± 3.2	-2.7 ± 2.7
				TRIS	-23.5 ± 2.0	-14.1 ± 1.6
			**ion. corrected**_._		**-53.9**	**15.9**
**5**	2 ± 1^*20*^	32 ± 9	-43.1 ± 0.8	HEPES	-50.2 ± 3.4	6.2 ± 3.7
				Tricin	-43.2 ± 4.1	0.5 ± 3.3
				TRIS	-34.3 ± 2.2	-8.3 ± 1.4
			**ion. corrected**		**-61.9**	**18.8**

The corresponding plot to get ionization corrected values is shown in Fig 9. Errors were estimated by means of standard deviation comprising at least triplicate measurements.

^[a]^ -TΔS_0_ was calculated according to the Gibbs-Helmholtz equation.

Compared to **1**, which lacks a substituent addressing the hydrophobic R34/P35-pocket, the thermodynamic profiles of **2** and **5** reveal very large favorable entropic contributions relative to **1** used as a reference (**1**→**2**: -TΔΔ*S*^*0*^ = -36.1 kJ∙mol^-1^, **1**→**5**: -TΔΔ*S*^*0*^ = -33.2 kJ∙mol^-1^). In contrast, the enthalpic signals of **2** and **5** are strongly decreased (**1**→**2**: ΔΔ*H*^*0*^ = 39.7 kJ∙mol^-1^, **1**→**5**: ΔΔ*H*^0^ = 31.7 kJ∙mol^-1^), which to some degree could be due to an enthalpy-entropy compensation as only a slight Δ*G*^*0*^ variation is observed [33]. As neither **2** nor **5** are able to exert any influence on dimer architecture, the changes in thermodynamic signature cannot arise from the collapse of the *loop-helix motif*, but only from the occupation of the R34/P35-pocket, the release of water molecules and a number of rearrangements of amino acid side chains in the vicinity of the R34/P35-pocket. As the binding of **2** and **5** involves the release of several previously well-bound water molecules compared to the binding pose of **1**, part of the entropic advantage of **2** and **5** over **1** can be explained. The desolvation of the 4-substituent, in case of **5** it even involves a charged group, will require an energy contribution which is supposedly of mainly enthalpic nature. This may explain the enthalpic loss of **2** and **5** in comparison to the unsubstituted reference **1**. The slight conformational adaptations observed near the R34/P35-pocket will likely show a mixed profile partitioning in enthalpic and entropic contributions.

Quite surprisingly, no detectable heat signal could be recorded for the interaction of **3** with TGT by directly titrating the ligand to the protein (S8 Fig). TGT∙**3**_co_ shows that this inhibitor occupies, similar to **2**, the R34/P35-pocket but, in addition, introduces huge structural rearrangements of the protein thereby destabilizing dimer formation (Fig 3a and 3b). If in an ITC experiment no heat signal is recorded, either no binding occurs or the binding event is entirely entropic. The first appears unlikely as we could determine submicromolar binding in our radio-labeled assay and the crystal structure proves binding [23]. Since any binding event in biological systems becomes enthalpically more favored with increasing temperature [34], we recorded the ITC titrations at higher temperature up to 308 K and we performed the titrations in HEPES buffer as the superimposed protonation change has the most exothermic contribution under these conditions. Nonetheless, in all titration experiments no heat signal could be detected for **3**. As a next step, we tried displacement titrations using the strong enthalpic binder **1** as displacement ligand. This strategy followed the idea that the known binding profile of **1** will be affected by the properties of ligand **3** being displaced in the titration experiment. Consulting our radio-labeled assay, the affinity of **1** is only by a factor of 6 more potent than **3**. Usually a much larger difference is required for meaningful displacement titration. We started with a solution in which TGT was 100% saturated with **3** and used **1** as titrant. Unfortunately, however, again no heat signal was recorded (S8 Fig). In three subsequent titrations, we reduced the saturation of TGT with **3** to about 90% and obtained measurable heat signals for binding of **1**. These experiments suggest two findings. First, the binding constant of **3** falls close to that of **1** in the ITC experiments. Second, the binding of **3** is supposedly overwhelmingly entropy-driven as even at high access of **1** the experiments did not result in a measurable signal. It is known that entropy-driven binders lead to difficulties in ITC measurements [35]. As indicated by TGT∙**3**_co_,we have to keep in mind that during binding and thus also titration, the disorder of the studied system including the ligand, the surrounding solvent, any protonation effects and changes in the dimer interface all contribute to the observed signal [34, 36]. It is likely that the binding signal of **3** is largely determined by the substantial rearrangements involving disorder phenomena of the *loop-helix motif* and residues across the dimer interface. Although we were not able to measure the thermodynamic parameters of the interaction between TGT and **3**, the entropic contribution (-TΔ*S*^*0*^) must be large to entirely compensate the enthalpic contribution so that the affinity is exclusively determined by the entropic contribution. The result is the more surprising, as the difference between both inhibitors **2** and **3** solely consists in a single methyl group, which is present in **3** but not in **2** and both inhibitors are of similar potency. The costs for desolvating **2** and **3** are assumed to be quite similar.

## Conclusions

In the course of a preceding study, we had synthesized the *lin*-benzoguanine derivatives **2** and **3**, which both are substituted with a furanoside moiety at position 4 and solely differ by a single methyl group attached to the 3´-OH function of **3**. The co-crystal structures TGT·**2**_co_ and TGT·**3**_co_ disclose that **3** but not **2** obviously takes influence on the architecture of the TGT dimer interface. This is reflected by the strongly differing thermodynamic binding signatures of **2** and **3**, revealing **2** as a more enthalpically favored binder whereas the equipotent **3** supposedly exhibits a pure entropically-driven binding signature in solution. TGT·**3**_co_ reveals the collapse of the so-called *loop-helix motif*, an important component of the dimer interface [24, 25], which obviously results in a considerable reduction of the *a*-axis and β-angle of the crystal unit cell. Consistent with the results of a previous study performed with ligand **4**, neither any influence of **3** on the dimer interface nor on unit cell parameters are observed in TGT·**3**_soak_.

This prompted us to revisit several of our previously investigated ligands (**5** to **9**) to assess their potential influence on the dimer interface. As they had only been investigated by soaking before [19, 27], we determined the co-crystal structures of these ligands in complex with TGT in this study. The new structures were systematically compared with those obtained by soaking. Except for **5**, all reinvestigated ligands clearly affect the dimer architecture of bacterial TGT. Their 4-substituents deeply penetrate into the R34/P35-pocket thereby bearing against its distal end, which leads to a considerable shift of Thr47. As Thr47 constitutes the first residue of the *loop-helix motif*, its displacement causes the collapse of this motif. In the case of **4**, the spiking needle-like 4-substituent directly contacts Thr47 and pushes it to the side [22]. In contrast, **6** does not directly address Thr47 but displaces the close-by Val45 belonging to the *hydrophobic floor* of the R34/P35-pocket. The Val45 shift entails a pronounced movement of the succeeding residues Gly46 and Thr47 leading to the breakdown of the *loop-helix motif*. In the case of ligands **3**, **7**, **8** and **9** the displacement of Thr47 occurs in a still more indirect manner as their 4-substituents initially contact Leu68, another component of the *hydrophobic floor* and shift this residue toward Val45. Consequently, Val45 is displaced, which again entails a large movement of Gly46 and Thr47.

The co-crystal structures TGT∙**5**_co_ and TGT∙**6**_co_ reveal that the 4-substituent has to exhibit certain spatial requirements to exert pressure on the distal end of the R34/P35-pocket and cause the movement of Thr47. Ligands **5** and **6** are highly similar and differ solely by a terminal cyclopentyl (**5**) or cyclohexyl ring (**6**). Obviously, the cyclopentyl of **5** perfectly fits into the R34/P35-pocket allowing deep immersion into this pocket without any expansion of its distal end. Accordingly, Thr47 is not displaced from its original position and the *loop-helix motif* remains unaffected. The 4-substituent of **6** also deeply penetrates into this pocket but the accommodation of the slightly more spacious cyclohexyl moiety requires a shift of Val45 involving the displacement of Thr47 and thus inducing the breakdown of the *loop-helix motif*.

The lipophilicity of the 4-substituent seems to be a further critical feature regarding the ability to impair proper TGT dimer interface formation. This becomes obvious upon the comparison of TGT∙**2**_co_ and TGT∙**3**_co_. The increased lipophilicity caused by the methylation of the 4-substituent´s 3´-OH group in **3** allows it to penetrate more deeply into the R34/P35-pocket and form van-der-Waals interactions with its *hydrophobic floor*. Thereby, it causes an expansion of the binding pocket ultimately leading to the collapse of the *loop-helix motif*. In contrast, the slightly more hydrophilic 4-substituent of **2** is not able to form equivalent interactions. Consequently, it adopts a more solvent-exposed conformation, which strongly differs from that of **3**. Thereby, it neither contacts the *hydrophobic floor* nor Thr47 and the *loop-helix motif* as well as the dimer interface remains intact.

Compared to TGT∙**1**_soak_, most soaked and co-crystallized complexes considered in this study reveal a shift of the Leu68-to-His73 stretch. This leads to a slight expansion of the R34/P35 binding pocket, required to fully accommodate the 4-substituent. TGT∙**3**-**4**_soak_ and TGT∙**6**-**9**_soak_ do not show, however, the major ligand-induced changes in dimer architecture, which only become obvious in the corresponding co-crystal structures. Obviously, within the lattice of a pre-formed apo-TGT crystal, the geometry of the *loop-helix motif* is largely constrained so that a ligand, which causes its breakdown in solution, is not able to induce such changes in pre-crystallized TGT. Instead, the 4-substituents of these ligands do not invade as deeply into the R34/P35-pocket as observed in the corresponding co-crystal structures. Therefore, energetically favorable interactions between the ligand´s 4-substituents and the protein cannot establish to full extent and the substituents become more flexible and badly defined in the electron density. In some cases, two conformations of the 4-substituent, accompanied by two conformations of the above-mentioned Leu68-to-His73 stretch (TGT∙**3**_soak_, TGT∙**8**_soak_, TGT∙**9**_soak_), become visible. Hereby, one of the two courses observed for the named stretch is highly similar to the one in complex with the 4-unsubstituted ligand **1** and forces the 4-substituent into a more solvent-exposed conformation. In contrast, in all co-crystal structures both the ligand´s 4-substituent and the Leu68-to-His73 stretch are observed in one conformation with the deeply buried 4-substituent being well-defined in the electron density.

**Fig 10:**
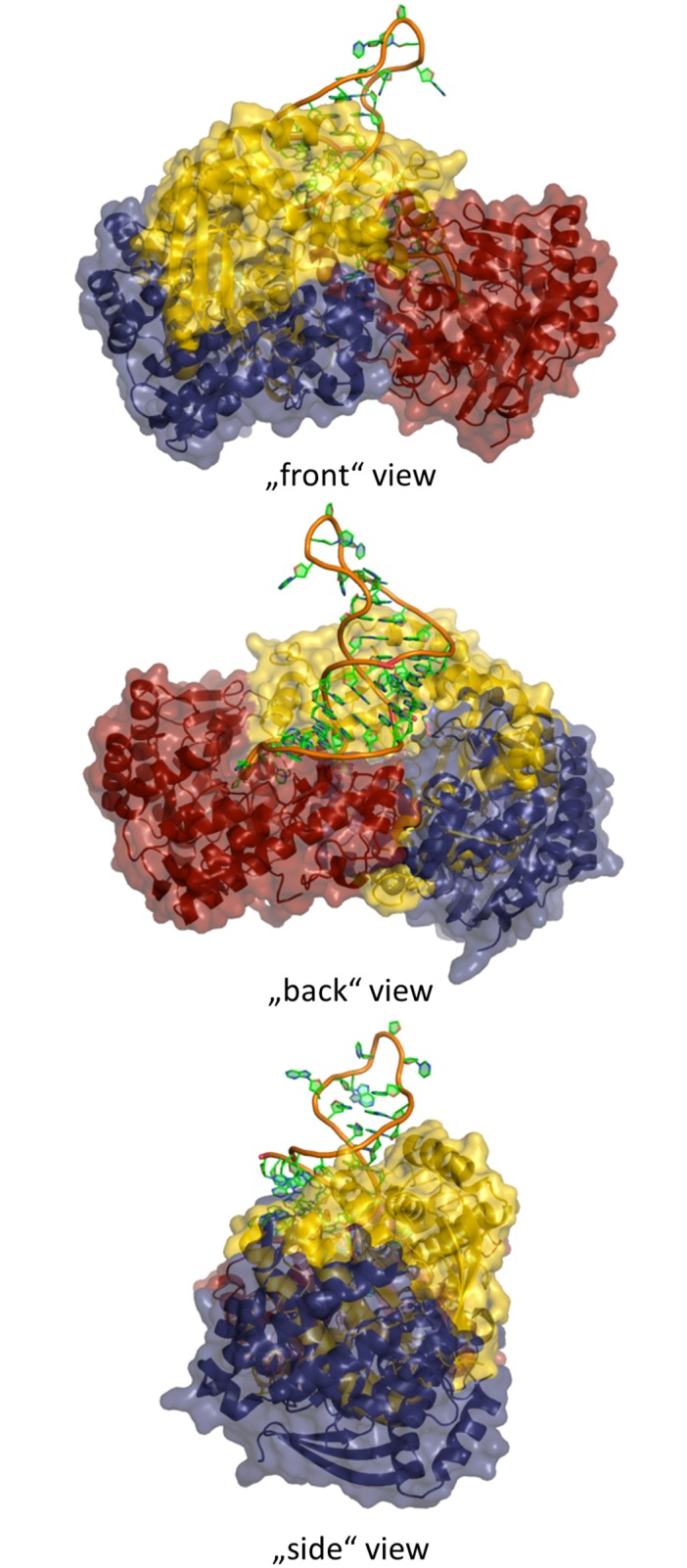
Alignment of TGT∙RNA (PDB entry: 1Q2R [14]) and twisted dimer found in TGT∙9_co_. The protein is represented as solvent accessible surface and cartoon with the following color code: twisted dimer: Monomer 1 red, Monomer 2 yellow. TGT∙RNA: Monomer 1 adopts a very similar orientation in space as in the twisted dimer (for the sake of clarity not shown in the figure). Monomer 2 blue. RNA: backbone orange and nucleosides green sticks.

Surprisingly, the binding of ligand **9** to TGT does not interfere solely with the geometry of the *loop-helix motif* but induces a total packing rearrangement of both TGT subunits. The resulting *twisted dimer*, never been observed before, displays a subunit contact area which is virtually identical in size to that observed for the conventional homodimer. Although a physiological role for this newly discovered dimer packing of TGT may not be excluded, it is clearly incompatible with the catalytic reaction performed by the enzyme. The crystal structure of *Z*. *mobilis* TGT in complex with an RNA substrate (PDB entry: 1Q2R [14]) displays the conventional dimer exclusively observed in TGT crystal structures up to now. For sterical reasons it is, although endowed with two active sites, only able to bind and convert one tRNA molecule at a time [14, 15, 19]. Thereby, one monomer fulfils catalysis, while the second supports the binding and orientation of the tRNA substrate through its *β*E*β*F loop. In the *twisted dimer*, this loop is involved in dimer interface formation and, consequently, not available for tRNA binding (Figs 6b and 7b). Furthermore, if the tRNA substrate bound to one subunit of the *twisted dimer* in the orientation required for catalysis it would inevitably lead to steric clashes with the second subunit (Fig 10).

Therefore, the *twisted dimer* most likely constitutes a non-productive state of the target enzyme, whose induction and/or stabilization may offer promising opportunities for further drug design. To this end, a cavity which is formed between the two subunits of the *twisted dimer* might prove useful. It is lined by the N-terminal end of loop *β*1*α*1, loop *α*E*α*F, the N-terminal part of helix *α*F and Arg286 within helix *α*B of both subunits and connects the two R34/P35-pockets of the *twisted* but not of the conventional dimer (S9 Fig).

## Materials and methods

### Preparation of recombinant *Z*. *mobilis* TGT

Expression of the *Z*. *mobilis tgt* gene in *Escherichia coli* using a plasmid based on vector pPR-IBA2 (IBA) was done as described previously [24, 25]. Cell disruption was achieved *via* an EmulsiFlex-C5^™^ high-pressure homogenizer (Avestin Europe GmbH) in 100 mL lysis buffer (20 mM TRIS pH 7.8, 10 mM EDTA, 1 mM DTT and 2 c*O*mplete^™^-Protease Inhibitor Cocktail Tablets (Roche) per 4 L of bacterial culture). Afterwards, the cell extract was centrifuged at 30.000 × g at 277 K for 1 hour. The clear supernatant was then loaded onto a Q-Sepharose Fast Flow anion exchange column (XK 26/15; GE Healthcare) conditioned with buffer A (10 mM TRIS pH 7.8, 1 mM EDTA, 1mM DTT). Subsequently, the column was washed with buffer A followed by the elution of the target protein by applying a linear gradient from 0 to 100% (v/v) buffer B (buffer A plus 1 M NaCl). The fractions containing TGT, endowed with an N-terminal Strep-tag II^®^, were then loaded onto a Strep-Tactin^®^ Superflow^®^ column (XK 16/10; IBA) conditioned with buffer W (100 mM TRIS pH 7.8, 1 M NaCl, 1 mM EDTA). After the column had been washed with buffer W, the protein was eluted with buffer E (buffer W plus 2.5 mM d-desthiobiotin). All chromatographic steps were carried out at room temperature using an ÄKTA purifier LC system (GE Healthcare). The TGT-containing fractions were then concentrated to approximately 2 mg∙mL^-1^ in high salt buffer (10 mM TRIS pH 7.8, 2 M NaCl, 1 mM EDTA) *via* VIVASPIN^®^20 centrifugal concentrators (Sartorius; 30,000 MWCO). Subsequently, the Strep-tag^®^ II was chipped off and separated from the target protein by means of the Thrombin Cleavage Capture Kit (Novagen^®^) following the manufacturer´s instructions. Thereby, 2.5 U of biotinylated thrombin per mg target protein was used during an incubation time of 16 to 18 hours at 293 K. Finally, the sample was dialyzed against high salt buffer and concentrated *via* VIVASPIN^®^20 centrifugal concentrators to 12 mg∙mL^-1^.

### *Z*. *mobilis* TGT crystallization

*Z*. *mobilis* TGT crystals were grown at 291 K using the hanging-drop vapor diffusion method. On a cover slip 1.5 μL of a 12 mg∙mL^-1^ *Z*. *mobilis* TGT solution (high salt buffer; see above) containing 1.6 mM of the respective inhibitor (stock solution in 100% DMSO) was mixed with the same volume of reservoir solution (100 mM MES pH 5.5, 1 mM DTT, 13% (w/v) PEG 8000, 10% (v/v) DMSO) and incubated in the presence of 650 μL reservoir solution. Crystals of appropriate size grew within a few days up to three weeks.

Inhibitor **3** was soaked into a pre-grown crystal of apo-TGT. For this purpose, *Z*. *mobilis* TGT crystals were grown as described above but in the absence of any inhibitor. On a cover slip suitable crystals of apo-TGT were then transferred into a droplet of 3 μL reservoir solution mixed with inhibitor stock solution to a final inhibitor concentration of 3.3 mM. In a hanging drop device the droplet was finally incubated in the presence of 650 μL reservoir solution for 24 h.

Prior to data collection, crystals were transferred into cryo-buffer (50 mM MES pH 5.5, 0.5 mM DTT, 300mM NaCl, 2% (v/v) DMSO, 4% (w/v) PEG 8000, 30% (v/v) glycerol) for several seconds and vitrified in liquid nitrogen.

### Data collection and processing

Diffraction data were collected under cryo-conditions (100 K) at BESSY MX beamline 14.1 (Inhibitor **7** and **8**), BESSY MX beamline 14.3 (Inhibitor **3** and **5**), ELETTRA XRD1 (Inhibitor **6**) and DESY P14 (Inhibitor **9**) with synchrotron radiation at wavelengths listed in Table 1.

All diffraction images were indexed, processed and scaled using XDS [37]. With the program XDSCONV the reflection data file was converted to mtz format and a subset of randomly selected 5% of all reflections were omitted for calculation of R_free_. *Z*. *mobilis* TGT usually crystallizes in monoclinic space group *C*2 containing one monomer per asymmetric unit with Matthews coefficients [38, 39] of 2.3 to 2.4 Å^3^∙Da^-1^. In the case of TGT∙**9**_co_, *Z*. *mobilis* TGT crystallized in space group *P*2_1_ with two monomers per asymmetric unit and a Matthew coefficient of 2.6 Å^3^∙Da^-1^. Data collection and refinement statistics are summarized in Table 1. Unit cell dimensions are given in Fig 2.

### Structure determination, refinement and analysis

Crystal structures were determined by molecular replacement using the program Phaser MR implemented in the CCP4 program suite.[40] As search model, the PDB entry 1P0D was used [18]. For TGT∙**9**_co_, amino acids between 45–68 as well as 280–291 were removed from the search model to find an appropriate solution. In between refinement cycles using Phenix.refine (version 1.10.1–2155_1492) [41], Coot [42] was used for model building. Initially five cycles of rigid body refinement were applied followed by five cycles of Cartesian simulated annealing. Further refinement cycles comprise coordinate xyz, occupancy, individual B-factor refinement and metal restraints for the zinc ion. For TGT∙**3**_soak_, TGT∙**5**_co_, TGT∙**6**_co_ and TGT∙**7**_co_ the *B*-factors of all atoms were anisotropically refined while for TGT∙**8**_co_ and TGT∙**9**_co_ only the zinc ion was anisotropically refined. For TGT∙**8**_co_ and TGT∙**9**_co_ TLS refinement was performed after selecting appropriate TLS groups with the TLS Motion Determination Server (TLSMD) [43]. Smiles codes of the inhibitors were obtained from www.molinspiration.com. For energetic minimization, coordinate- and restraint-file generation the Grade Web Server was used [44]. Alternate conformations of side chains, missing amino acids in the search model, water molecules and inhibitor atoms, were fitted into well-defined positive m|*F*_o_|–D|*F*_c_| electron density and were kept during refinement if the corresponding 2|*F*_o_|–|*F*_c_| electron density was sufficient and the occupancy exceeded 20% (alternate conformations of side chains or inhibitor atoms). For adding initial water molecules, the option “Find Waters” included in Coot was used. Only those water molecules which were, after refinement, characterized by a 2|*F*_o_|–|*F*_c_| electron density peak with σ ≥ 1.0 RMSD, a *B*-factor not significantly larger than 55 Å^2^ and distances between neighboring atoms from 2.3 Å to 3.5 Å were kept in the structures. For the soaking structure of **3** (TGT∙**3**_soak_), the occupancy for the furanosyl moiety was refined in a constrained group with the alternative group A (residues Leu68 to His73, final occupancy = 57%) against the alternative group B (residues Leu68 to His73, final occupancy = 43%) to avoid a steric clash between the furanosyl moiety and the alternative group B. In the case of TGT∙**8**_co_ the occupancy of the toluyl side chain of the inhibitor was refined as a group to a final value of 63%. For the identification of chloride ions anomalous maps were calculated with the program ANODE [45]. In the complex TGT∙**5**_co_, an anomalous density signal of σ = 5.4 appeared and was modeled as chloride ion. The chloride ion in the co-crystal TGT∙**6**_co_ has an anomalous density signal of σ = 4.5. All figures displaying crystal structures were produced using PyMOL [46]. Hydrogen bonds, salt bridges and van-der-Waals contacts were assigned with the program CONTACTSYM [47, 48]. The cutoff for hydrogen bonds and salt bridges was 3.7 Å and up to 4.2 Å for van-der-Waals contacts, depending on atom type and using standard van-der-Waals contacts. The contact surface of the two monomers forming the homodimer was calculated by PDBePISA [49]. RMSD calculation was performed using the McLachlan algorithm as implemented in the program ProFit (http://www.bioinf.org.uk/software/profit/) [50].

### Determination of inhibition constants

The inhibition constant (*K*_i_) of ligand **4** (competitive inhibition with respect to tRNA substrate) was determined as described by Meyer *et al*. (2006) [30] with slight modifications: The used assay buffer contained 100 mM HEPES pH 7.3, 20 mM MgCl_2_ and 0.037% (v/v) Tween 20. The concentration of *Z*. *mobilis* TGT amounted to 9 nM, [8-^3^H]-guanine was used at a (saturating) concentration of 10 μM and tRNA^Tyr^ at a concentration of 1.5 μM. Aliquots of 15 μL were withdrawn for scintillation counting every 60 minutes (in total four).

The *K*_i_ value was averaged over three measurements. The preparation of *E*.*coli* tRNA^Tyr^ (ECYC2) [51] *via in vitro* transcription was carried out using the RiboMAX^™^ Large Scale RNA Production System-T7 (Promega) according to the vendor´s protocol.

### Native MS experiments

Nano-ESI-MS analyses were performed as already described in the literature [22, 24, 25]. Briefly, WT-TGT samples were exchanged twice against a 1 M ammonium acetate pH 7.5 buffer using microcentrifuge gel-filtration columns (Zeba 0.5 mL, Thermo Scientific). Next, protein concentration was determined spectrophotometrically at 280 nm (Nano-Drop 2000 spectrophotometer, Thermo Scientific).

Native MS experiments were carried out on a hybrid Q-TOF mass spectrometer (Synapt G2 HDMS, Waters) equipped with an automated chip-based nanoESI source (Triversa Nanomate, Advion Biosciences) operating in the positive ion mode. External calibrations were generated from the multiple charged ions produced by a 2 μM horse heart myoglobin solution diluted in a 1:1 (v/v) water: acetonitrile mixture acidified with 1% (v/v) formic acid (denaturing MS) and singly charged cesium iodide clusters diluted to 2 g∙L^-1^ in a 1:1 (v/v) water: isopropanol solution (native MS).

Protein homogeneity and purity was first assessed under denaturing conditions (TGT monomer 2 μM, 1:1 (v/v) water: acetonitrile mixture acidified with 1% (v/v) formic acid) using standard interface tuning parameters (cone voltage V_c_ and backing pressure P_i_, set to 40 V and 2.1 mbar, respectively).

Titration experiments in native conditions (1 M NH_4_Ac pH 7.5) were next triggered by mixing 2.5 μM TGT monomers with 25 μM of each ligand separately (ratio 1:10). The final DMSO concentration of each mixture never exceeded 0.25% (v/v). Non-covalent TGT dimers and TGT-ligand complexes were preserved in the gas phase according fine-tuned instrumental parameters, improving ion desolvation and transmission while maintaining intact structures (V_c_ = 80V, P_i_ = 6 mbar).

Relative quantification of TGT monomers was performed from charge states peak intensities of both monomeric (10 to 14+) and dimeric complexes (15 to 20+), using MassLynx 4.1 (Waters). TGT dimer disruption was determined for each ligand through their destabilization factor (Fig 8) which corresponds to their associated ratio of TGT monomers normalized from the apo-condition (S7 Fig; S1 Table). Standard deviations are calculated here from analytical triplicates. For the sake of reliability and comparability, all these experiments were performed within the same day, from identical experimental and instrumental conditions using the same buffer exchanged TGT batch (frozen and thawed only once).

### Isothermal titration calorimetry

ITC measurements were performed using a Microcal ITC200 calorimeter system from GE Healthcare. *Z*. *mobilis* TGT was dissolved in the ITC buffer (50 mM HEPES/Tricin/TRIS pH 7.8, 200 mM NaCl, 0.037% (v/v) Tween 20 and 3% (v/v) DMSO) to a final concentration of 10–20 μM.

The inhibitors were first dissolved in 100% (v/v) DMSO and diluted with the buffer solution to a final DMSO concentration of 3% (v/v). The concentration of the inhibitor in the syringe was adjusted to 200–300 μM with the ITC buffer. The reference cell contained filtered demineralized water. All ITC experiments were performed at 298 K with a reference power of 5 kcal∙s^-1^. The initial delay before the injections were started was adjusted to 180–300 s and the spacing between each injection to 150–180 s. The first injection contained 0.3–0.6 μL of the inhibitor solution followed by injections of 1.0–2.4 μL until a saturation occurred. A stirring speed of 750 rpm was chosen. Raw data were collected as released heat per time. The direct titrations were done in three different buffers (HEPES/Tricin/TRIS) to correct for the overlaid protonation effect as previously described [29, 52].The measured Δ*H*^*0*^_obs_ were plotted on the ordinate and the ionization enthalpy of the corresponding buffer Δ*H*^*0*^_ion_ [53] on the abscissa (Fig 9). The positive slope of the linear regression line indicates how many protons per mole of formed complex are entrapped. The interception with the ordinate reveals the enthalpy corrected for the protonation effect (Δ*H*^*0*^_bind_).

For inhibitor **3**, displacement titrations in HEPES have been attempted, as all titrations in the three applied buffers revealed no suitable heat signal [54, 55]. For estimation of binding affinity (*K*_d_), *Z*. *mobilis* TGT was incubated with 90–100% saturation of inhibitor **3**. Protein saturation was calculated according Rühmann *et al*. (2015) [56]. Inhibitor **1** as strong titrant was used to displace inhibitor **3**.

For analysis of the raw data, peak integration, model fitting and evaluation, the programs NITPIC [57] and SEDPHAT [58] were used. The initial injection peak was removed and the areas of the remaining peaks were integrated. The correction of the heats of dilution was conducted by subtraction of the final constant injection peak area integrals. The A+B ↔ AB hetero-association model (1:1 stoichiometry) was applied, whereby Δ*H*^*0*^ and *K*_d_ were directly obtained. The Δ*G*^*0*^ and -T*ΔS*^0^ values were calculated using a temperature of 298.15 K. For illustration of the integrated raw data and the ITC isotherms, the program GUSSI was used [59]. Obtained thermodynamic data per buffer are reported as mean values averaged over at least three measurements.

### Synthesis of inhibitors

Inhibitors were synthesized and purified as described in detail elsewhere [22, 23, 26, 30].

#### Alignment and figures

Figures were prepared using ChemDraw Std 12.0 (PerkinElmer, Massachusetts, USA) and Pymol.

#### Protein data bank accession codes

Coordinate files of all newly reported crystal structures were deposited in the PDB and are available upon article publication under the accession codes: 5I09 (TGT∙**3**_soak_), 5I00 (TGT∙**5**_co_), 5I02 (TGT∙**6**_co_), 5I06 (TGT∙**7**_co_), 5I03 (TGT∙**8**_co_) and 5I07 (TGT∙**9**_co_).

## Supporting information

S1 Fig Active site of apo-TGT and binding modes of inhibitor 1 in TGT∙1_soak_ and 2 in TGT∙2_co_. (PDF)

S2 Fig Binding modes of inhibitor 4 in corresponding soaking and co-crystal structures. (PDF)

S3 Fig Binding modes of inhibitors 3, 5, 6 in corresponding soaking and co-crystal structures. (PDF)

S4 Fig Binding modes of inhibitors 7, 8, 9 in corresponding soaking and co-crystal structures. (PDF)

S5 Fig Captured chloride ion. (PDF)

S6 Fig Incorporated water molecules around Thr47 of the *β1α1*-loop. (PDF)

S7 Fig S1 Tab. Relative proportions of TGT monomers measured by native MS.
(PDF)

S8 Fig Representative thermograms and fitted regression curves (ITC). (PDF)

S9 Fig Cavity inside the interface of the twisted dimer in TGT∙9_co_. (PDF)

S1 Table (PDF)
