# Supplementary material for: Soaking suggests “alternative facts”: Only co-crystallization discloses major ligand-induced interface rearrangements of a homodimeric tRNA-binding protein indicating a novel mode-of-inhibition
Source: PLoS One. 2017 Apr 18;12(4):e0175723. doi: 10.1371/journal.pone.0175723 (PMC5395182; doi:10.1371/journal.pone.0175723)
Supplement: S1 Fig — (PDF) [file pone.0175723.s001.pdf]

Active site of apo-TGT and binding modes of inhibitor 1 in TGT·1_{soak} and 2 in TGT·2_{co}

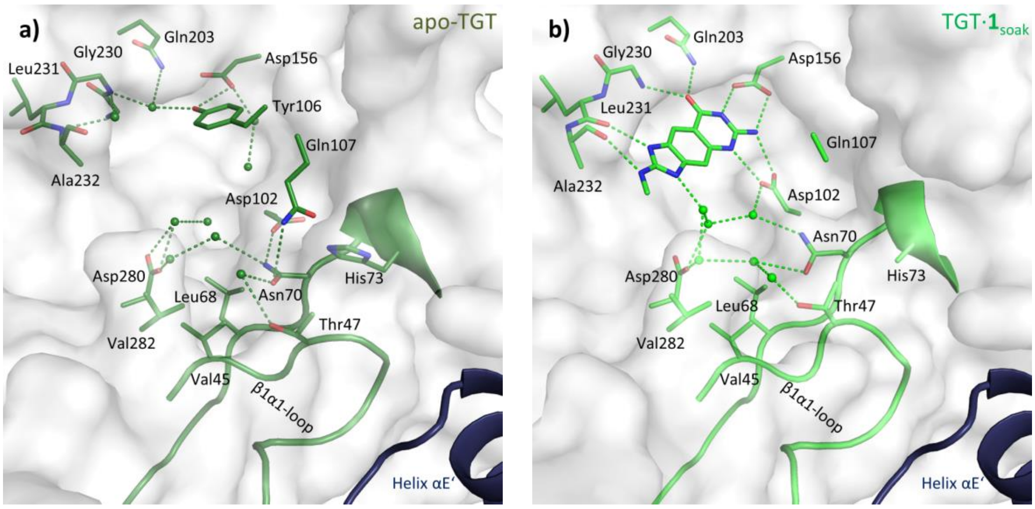

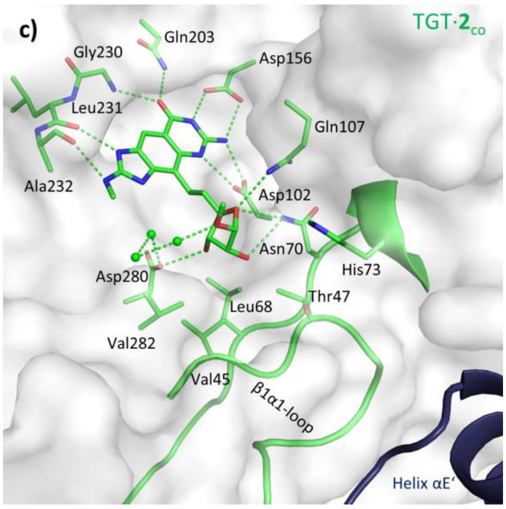

Figure S1. **a)** Active site of apo-TGT (carbons green; PDB entry: 1P0D) and binding modes of **b) 1** (carbons light green) in TGT·$\mathbf{1}_{soak}$ and **c) 2** in TGT·$\mathbf{2}_{co.}$ Selected water molecules are shown as spheres and colored as the corresponding complex. Oxygen red, nitrogen blue. For the sake of clarity Tyr106 and Met260 are not shown. Solvent accessible surface is colored in gray. Dashed lines indicate H-bonds (2.5 - 3.5 Å). Selected portions of the second monomer of the homodimer are shown as dark blue cartoon.
