# Supplementary material for: Soaking suggests “alternative facts”: Only co-crystallization discloses major ligand-induced interface rearrangements of a homodimeric tRNA-binding protein indicating a novel mode-of-inhibition
Source: PLoS One. 2017 Apr 18;12(4):e0175723. doi: 10.1371/journal.pone.0175723 (PMC5395182; doi:10.1371/journal.pone.0175723)
Supplement: S2 Fig — (PDF) [file pone.0175723.s002.pdf]

Binding modes of inhibitor 4 in corresponding soaking and co-crystal structures

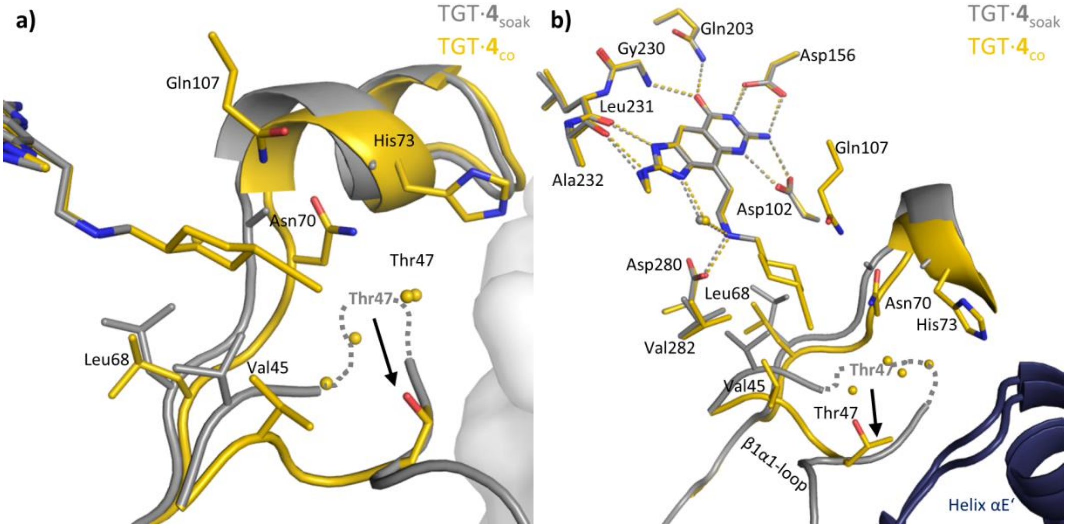

Figure S2. Comparison of binding modes and structural rearrangements of inhibitor **4**.TGT·**4**$_{soak}$ (carbons gray) and TGT·**4**$_{co}$ (carbons yellow). Selected water molecules are shown as spheres and colored as the corresponding complex. **a)** For the sake of clarity, residues of the guanine-34/preQ$_1$ binding site that interact with the *lin*-benzoguanine scaffold are not shown. Solvent accessible surface and selected residues as sticks of the second monomer of the homodimer are colored gray. Position of missing Thr47 in TGT·**4**$_{soak}$ is indicated as gray dashed lines. Black arrows indicate positional differences between the crystal structures of different crystallization protocols. **b)** Active site of TGT·**4**$_{soak}$ and TGT·**4**$_{co}$. Dashed lines indicate H-bonds (2.6 - 3.5 Å). Selected portions of the second monomer of the homodimer are shown as dark blue cartoon.
