# Supplementary material for: Soaking suggests “alternative facts”: Only co-crystallization discloses major ligand-induced interface rearrangements of a homodimeric tRNA-binding protein indicating a novel mode-of-inhibition
Source: PLoS One. 2017 Apr 18;12(4):e0175723. doi: 10.1371/journal.pone.0175723 (PMC5395182; doi:10.1371/journal.pone.0175723)
Supplement: S4 Fig — (PDF) [file pone.0175723.s004.pdf]

Binding modes of inhibitors 7, 8, 9 in corresponding soaking and co-crystal structures

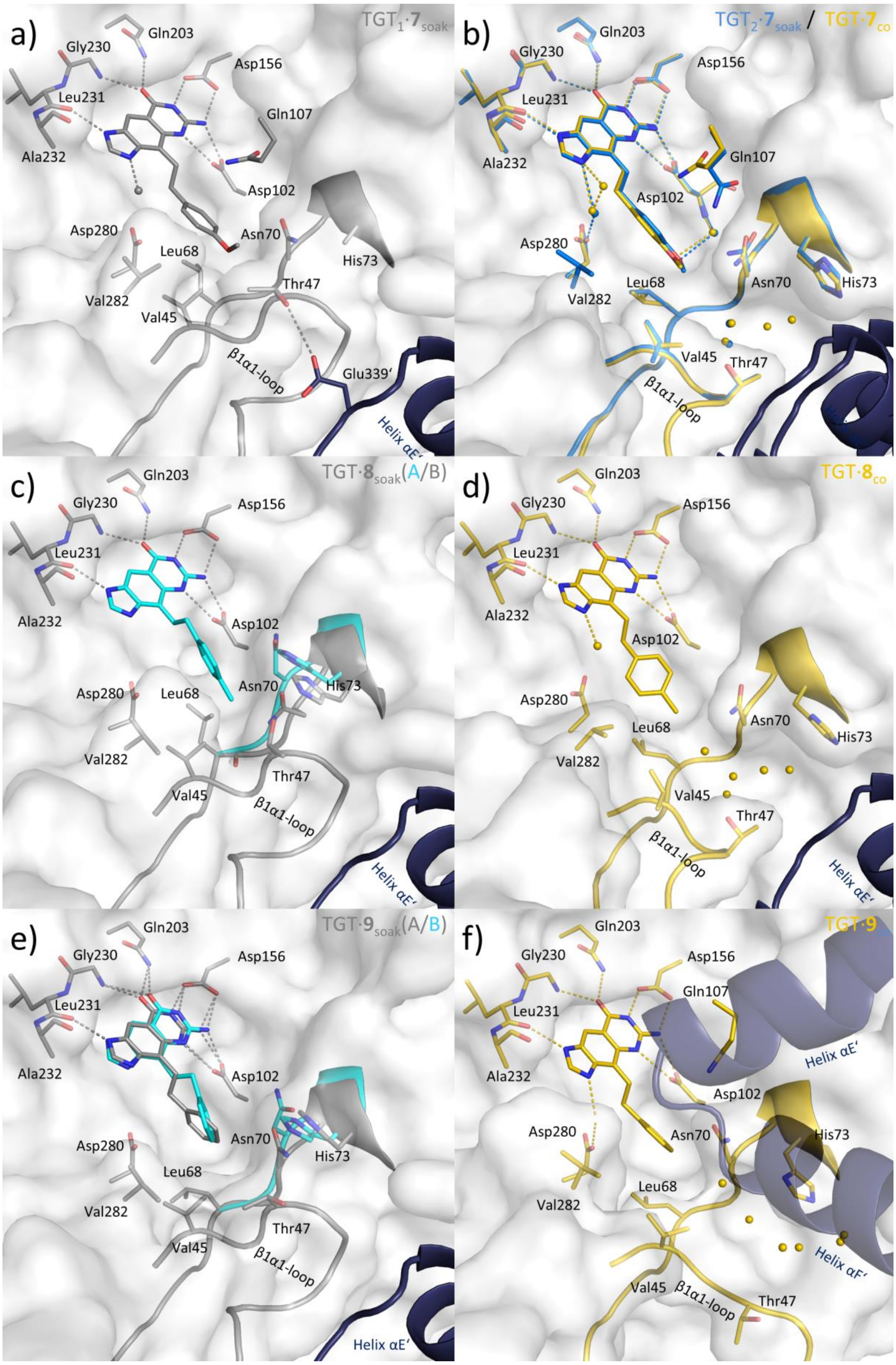

Figure S4. Active site of **a)** TGT$_1$·**7**$_{soak}$ (carbons gray), **b)** TGT$_2$·**7**$_{soak}$ (carbons blue) & TGT·**7**$_{co}$ (carbons yellow), **c)** TGT·**8**$_{soak}$ (carbons gray and cyan, indicating the split conformations observed in the protein), **d)** TGT·**8**$_{co}$ (carbons yellow), **e)** TGT·**9**$_{soak}$ (carbons gray and cyan, indicating the split conformations observed for the protein and ligand), **f)** TGT·**9**$_{co}$ (carbons yellow). Selected water molecules are shown as spheres and colored as the corresponding complex. Oxygen red, nitrogen blue. For the sake of clarity Tyr106 and Met260 are not shown. Solvent accessible surface is displayed in gray. Dashed lines indicate H-bonds (2.6 - 3.7 Å). Selected portions of the second monomer of the homodimer are shown as dark blue cartoon.
