# Supplementary material for: Soaking suggests “alternative facts”: Only co-crystallization discloses major ligand-induced interface rearrangements of a homodimeric tRNA-binding protein indicating a novel mode-of-inhibition
Source: PLoS One. 2017 Apr 18;12(4):e0175723. doi: 10.1371/journal.pone.0175723 (PMC5395182; doi:10.1371/journal.pone.0175723)
Supplement: S5 Fig — (PDF) [file pone.0175723.s005.pdf]

Captured chloride Ion

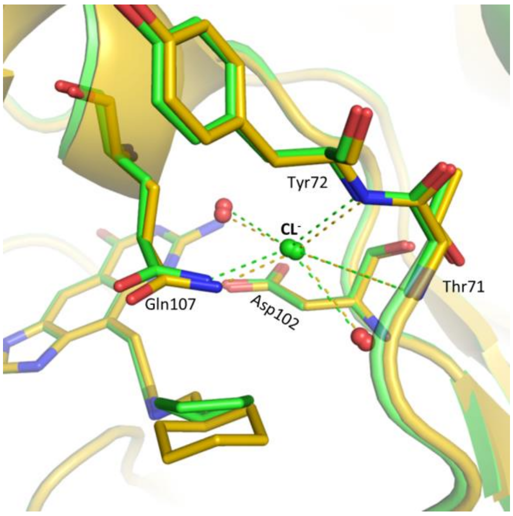

Figure S5. Position of a recurring chloride ion. Protein is displayed as cartoon. Chloride ions are presented as green spheres. Inhibitors and residues involved in the coordination of the chloride ion are presented as sticks. Selected water molecules are displayed as red spheres. The interactions of the chloride ions are between 3.1 and 3.8 Å to Gln107-NH, Thr71-NH, Tyr72-NH and to two water molecules. Coordination of the chloride ion in TGT·$\mathbf{5}_{co}$ (carbons green) is displayed as green dashed lines. The peak intensity of the anomalous signal is σ = 5.4. The chloride ion coordination in TGT·$\mathbf{6}_{co}$ (carbons yellow) is colored in yellow. The corresponding peak intensity of the anomalous signal is σ = 4.5. The chloride probably originates from the 2 M NaCl (high salt buffer) used for the storage of TGT.
