# Supplementary material for: Soaking suggests “alternative facts”: Only co-crystallization discloses major ligand-induced interface rearrangements of a homodimeric tRNA-binding protein indicating a novel mode-of-inhibition
Source: PLoS One. 2017 Apr 18;12(4):e0175723. doi: 10.1371/journal.pone.0175723 (PMC5395182; doi:10.1371/journal.pone.0175723)
Supplement: S6 Fig — (PDF) [file pone.0175723.s006.pdf]

Incorporated water molecules around Thr47 of the *β*1*α*1-loop

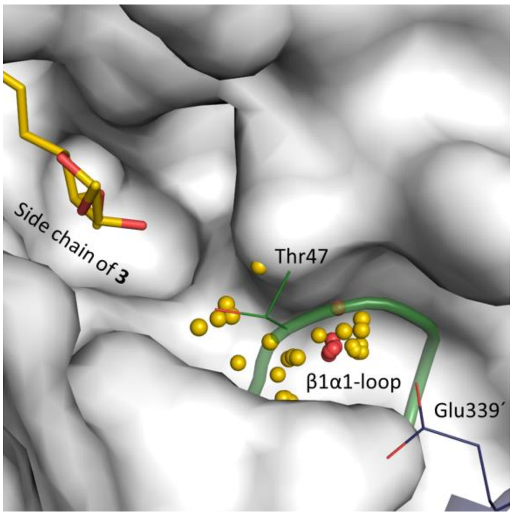

Figure S6. Incorporated water molecules around Thr47 of all investigated TGT·inhibitor complexes. Solvent accessible surface of TGT·**3**$_{co}$ is displayed in gray; inhibitor **3** is colored yellow and presented as sticks. Selected residues of apo-TGT structure (PDB entry: 1P0D [1]) are shown as lines or cartoon and are colored green. Residue Glu339 of the second monomer of the homodimer is colored dark blue. Water molecules around Thr47 of the *β*1*α*1-loop (as taken from apo-protein structure) are shown as spheres. Color coding for water molecules:

Yellow for complexes where the *β*1*α*1-loop was disturbed by ligand binding (TGT·**3**, **4**, **6** – **9**$_{co}$ & TGT$_2$·**7**$_{soak}$).

Red for complexes where the *β*1*α*1-loop was not disturbed by ligand binding (TGT·**1**$_{soak}$, TGT·**2**$_{co}$ and TGT·**5**$_{co}$).

Reference

1. Brenk R, Stubbs MT, Heine A, Reuter K, Klebe G (2003) Flexible adaptations in the structure of the tRNA-modifying enzyme tRNA-guanine transglycosylase and their implications for substrate selectivity, reaction mechanism and structure-based drug design. ChemBioChem 4: 1066-1077.
