# Supplementary material for: Soaking suggests “alternative facts”: Only co-crystallization discloses major ligand-induced interface rearrangements of a homodimeric tRNA-binding protein indicating a novel mode-of-inhibition
Source: PLoS One. 2017 Apr 18;12(4):e0175723. doi: 10.1371/journal.pone.0175723 (PMC5395182; doi:10.1371/journal.pone.0175723)
Supplement: S7 Fig — Relative proportions of TGT monomers measured by native MS. (PDF) [file pone.0175723.s007.pdf]

Relative proportions of TGT monomers measured by native MS

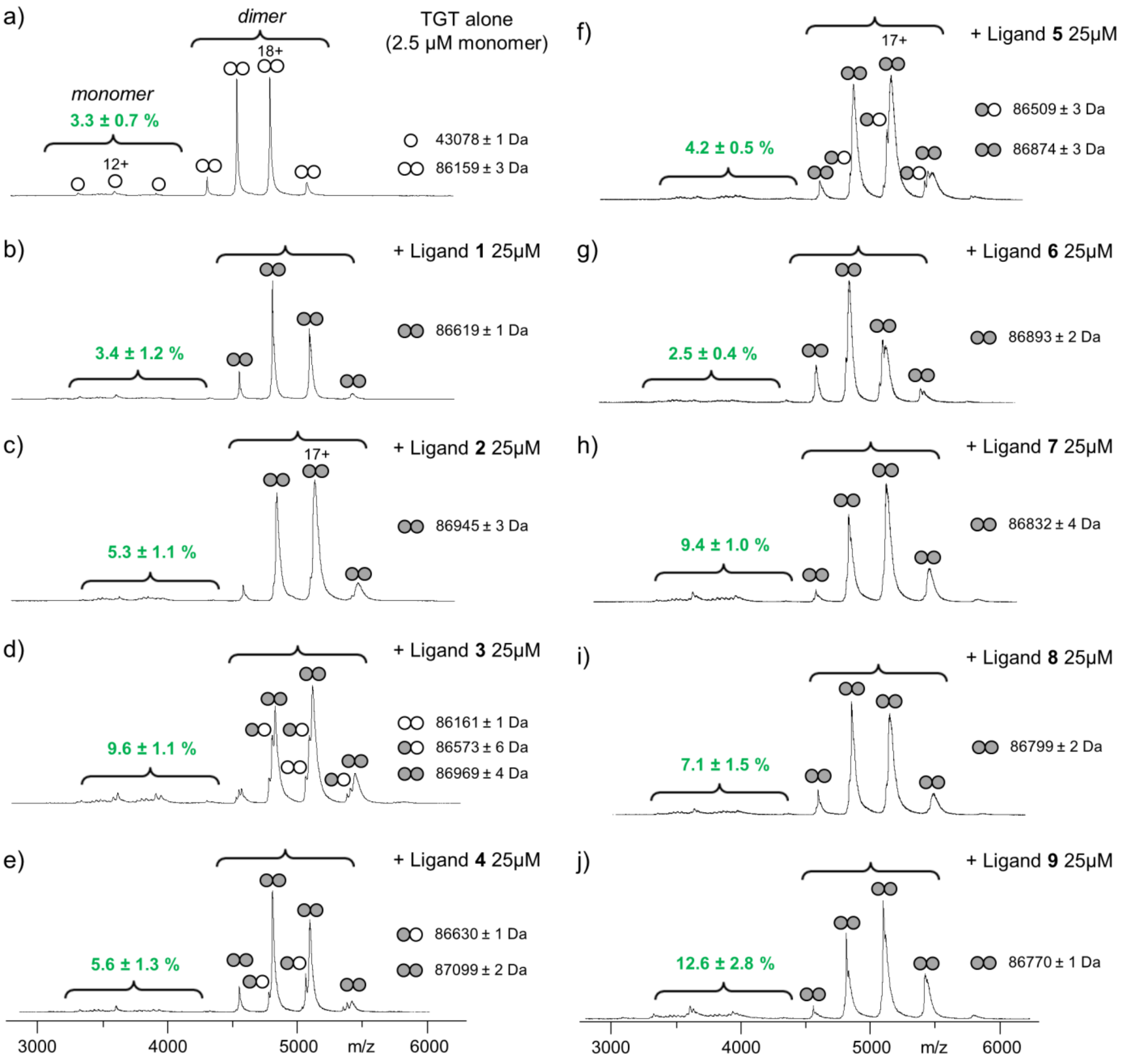

Figure S7. Native MS spectra of TGT:ligand mixtures. Measurements were performed in 1M NH_4Ac pH 7.5, V_c = 80V, P_i = 6 mbar. **a)** Native MS spectrum of apo-TGT (TGT monomers = 2.5 µM). **b) – j)** Native MS spectra of holo-TGT:ligand **1 – 9** mixtures (TGT monomers = 2.5 µM; Ligand = 25 µM).
