# Supplementary material for: Soaking suggests “alternative facts”: Only co-crystallization discloses major ligand-induced interface rearrangements of a homodimeric tRNA-binding protein indicating a novel mode-of-inhibition
Source: PLoS One. 2017 Apr 18;12(4):e0175723. doi: 10.1371/journal.pone.0175723 (PMC5395182; doi:10.1371/journal.pone.0175723)
Supplement: S8 Fig — (PDF) [file pone.0175723.s008.pdf]

Representative thermograms and fitted regression curves (ITC)

Inhibitor 1	Inhibitor 2	Inhibitor 3	Inhibitor 3	Inhibitor 5
(direct titration)	(direct titration)	(direct titration)	(displacement titration) [a]	(direct titration)

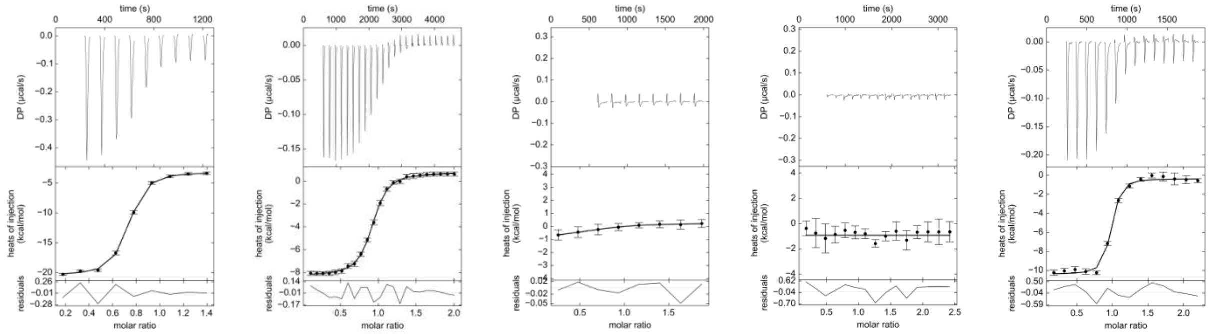

Figure S8. Representative thermograms and fitted regression curves in HEPES ITC buffer. [a]Protein saturation was calculated with $K_i \approx K_d$ as published.[1] Cell: 20 µM TGT incubated with 300 µM of **3** (100% saturation). Inhibitor **1** was used as titrant.

Reference

1. Rühmann E, Betz M, Fricke M, Heine A, Schäfer M, Klebe, G (2015) Thermodynamic signatures of fragment binding: validation of direct versus displacement ITC titrations. Biochim Biophys Acta 1850: 647-656.
