# Supplementary material for: Soaking suggests “alternative facts”: Only co-crystallization discloses major ligand-induced interface rearrangements of a homodimeric tRNA-binding protein indicating a novel mode-of-inhibition
Source: PLoS One. 2017 Apr 18;12(4):e0175723. doi: 10.1371/journal.pone.0175723 (PMC5395182; doi:10.1371/journal.pone.0175723)
Supplement: S9 Fig — (PDF) [file pone.0175723.s009.pdf]

Cavity inside the interface of the *twisted dimer* in TGT·9_{co}

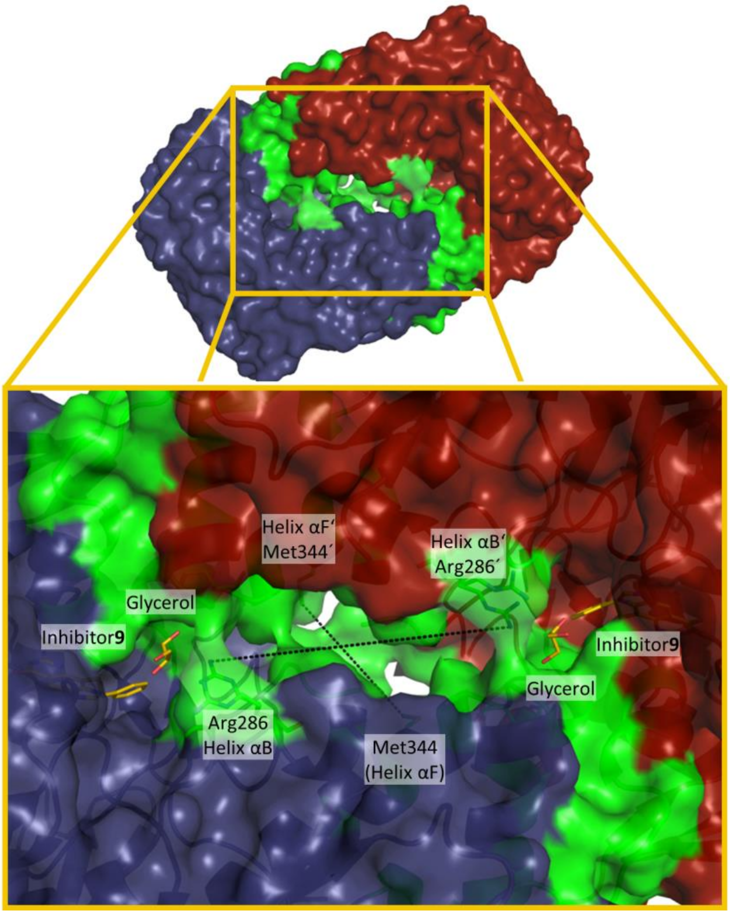

Figure S9. Overview of the cavity inside the *twisted dimer* interface found in TGT·**9**$_{co}$. The protein is displayed as cartoon and the surface is colored in red (monomer 1) and yellow (monomer 2). Residues involved in the dimer interface in both monomers, determined by PDBePISA [3], are colored in green. Glycerol and inhibitor **9** are represented as yellow sticks. Distances between Met344-Met344’ (11.8 Å) and Arg286-Arg286’ (21.5 Å) are displayed as black dotted lines.

Reference

1. Krissinel E, Henrick K (2007) Inference of macromolecular assemblies from crystalline state. J Mol Biol 372: 774-797.
