# Supplementary material for: Soaking suggests “alternative facts”: Only co-crystallization discloses major ligand-induced interface rearrangements of a homodimeric tRNA-binding protein indicating a novel mode-of-inhibition
Source: PLoS One. 2017 Apr 18;12(4):e0175723. doi: 10.1371/journal.pone.0175723 (PMC5395182; doi:10.1371/journal.pone.0175723)
Supplement: S1 Table — (PDF) [file pone.0175723.s010.pdf]

Table S1. Relative proportions of TGT monomers deduced from native MS study of TGT:ligand 1:10 mixtures

Relative proportion of TGT monomers (%) at 2.5 µM TGT and 25 µM Inhibitor (1:10)									
**Inhibitor**	**1**	**2**	**3**	**4**	**5**	**6**	**7**	**8**	**9**
1:10 ratio	3.4 ± 1.2	5.3 ± 1.1	9.6 ± 1.1	5.6 ± 1.3	4.2 ± 0.5	2.5 ± 0.4	9.4 ± 1.0	7.1 ± 1.5	12.6 ± 2.8

Measurements were performed in 1 M NH_4Ac pH7.5, V_c = 80V, P_i = 6mbar. For inhibitors **4** and **6**, partial precipitation upon ligand dilution in NH_4Ac buffer was detected. Analysis of 2.5 µM TGT in absence of any ligand resulted in 3.3 ± 0.7 % TGT monomer. Corresponding errors are here expressed from triplicate measurements.
